# Preoperative PET imaging and fluorescence-guided surgery of human glioblastoma using dual-labeled antibody targeting ET_A_ receptors in a preclinical mouse model: A theranostic approach

**DOI:** 10.7150/thno.98163

**Published:** 2024-09-30

**Authors:** Marie Hautiere, Delphine Vivier, Paul Dorval, Donovan Pineau, Dimitri Kereselidze, Caroline Denis, Amaury Herbet, Narciso Costa, Claire Bernhard, Victor Goncalves, Erwan Selingue, Benoit Larrat, Pierre Alix Dancer, Jean-Philippe Hugnot, Didier Boquet, Charles Truillet, Franck Denat

**Affiliations:** 1 Université Paris-Saclay, CEA, INRAE, Médicaments et Technologies pour la Santé (MTS), SPI, Laboratoire d'Etude de l'Unité Neurovasculaire et Innovation Thérapeutique (LENIT), 91191 Gif-sur-Yvette, France.; 2 Université de Bourgogne, ICMUB UMR CNRS 6302, Dijon, 21000, France.; 3 Kaer Labs, 44300 Nantes, France.; 4 Université Paris-Saclay, CEA, CNRS, Inserm, BioMaps, Orsay, 91401, France.; 5 Institut de Génomique Fonctionnelle, Université de Montpellier, CNRS, INSERM, Montpellier, France.; 6 Université Paris-Saclay, CEA, CNRS, NeuroSpin/BAOBAB, Gif sur Yvette, France.

**Keywords:** Fluorescence-Guided surgery, ImmunoPET, Endothelin A (ET_A_), Glioblastoma, Bimodal Imaging

## Abstract

**Rationale:** Glioblastoma (GBM) poses significant challenges regarding complete tumor removal due to its heterogeneity and invasiveness, emphasizing the need for effective therapeutic options. In the last two decades, fluorescence-guided surgery (FGS), employing fluorophores such as 5-aminolevulinic acid (5-ALA) to enhance tumor delineation, has gained attraction among neurosurgeons. However, some low-grade tumors do not show any accumulation of the tracers, and the lack of patient stratification represents an important limitation. Since 2000, endothelin axis has been extensively investigated for its role in cancer progression. More specifically, our team has identified endothelin A receptors (ET_A_), overexpressed in glioblastoma cancer stem cells, as a target of interest for GBM imaging. This study aims to evaluate the efficacy of a novel preclinical bimodal imaging agent, [^89^Zr]Zr-axiRA63-MOMIP, as a theranostic approach to: i) detect ET_A_^+^ cells in an orthotopic model of human GBM, ii) achieve complete tumoral resection.

**Methods:** Monomolecular multimodal imaging platform (MOMIP) - containing both a fluorophore (IRDye800CW) and a chelator for a positron-emitting radiometal (desferroxamine B, DFO) - was conjugated to the axiRA63 antibody targeting ET_A_ receptors, overexpressed on the surface of GBM stem cells. Mice bearing orthotopic human GBM were imaged 48 h post injection of [^89^Zr]Zr-axiRA63-MOMIP via positron emission tomography (PET) and optical imaging. Subsequently, post-mortem proof-of-concept FGS was implemented as well as *ex vivo* analyses (H&E staining, autoradiography, serial block face imaging) on brains with resected or unresected tumor to assess the correlation between PET and fluorescence signals.

**Results:** PET imaging of [^89^Zr]Zr-axiRA63-MOMIP enabled a clear detection of ET_A_^+^ cells in an orthotopic model of human GBM. Intraoperative optical imaging allowed a near-complete tumor resection together with the visualization of a weak fluorescence signal, after a prolonged exposure time, that was attributed to residual tumor cells *via* H&E staining. Besides, a qualitative correlation between the signals of both modalities was observed.

**Conclusions:** The use of [^89^Zr]Zr-axiRA63-MOMIP provides an effective theranostic approach to detect and treat GBM by surgery in a preclinical mouse model. Thanks to the high correlation between PET and fluorescence signal allowing patients stratification, this bimodal agent should have a great potential for clinical translation and should present a significant advantage over non-targeted fluorophores already used in the clinic.

## Introduction

Glioblastoma (GBM), classified as a CNS WHO grade 4 tumor among high-grade gliomas 1, stands as the most prevalent and aggressive primary brain tumor in adults and exhibits a complex array of pathological features. Glioblastoma presents substantial therapeutic challenges owing to its diverse nature and complexity 2. When a GBM is suspected following an MRI scan, a neurosurgeon assesses the feasibility of tumor removal based on its location and infiltration. If surgery is possible, this initial therapeutic approach is often crucial for the prognosis of the patient and can be extremely effective 3,4. However, margin delineation remains challenging for neurosurgeons and can significantly impact long-term survival - as residual tumor tissue often leads to recurrence - as well as patient's quality of life, since an excessive tumor resection could lead to neurological deficits. Over the last two decades, fluorescence-guided surgery (FGS) - a technique facilitating more comprehensive resection thanks to fluorescent tumor tissue - has gained significant interest and holds promise in promoting patient survival while adhering to the standard treatment protocol 5,6. 5-aminolevulinic acid (5-ALA) - a precursor in heme synthesis approved by the EMA in 2007 and FDA in 2017 7 - ranks among the most investigated optical imaging (OI) agents for brain surgery. Thanks to its good penetration of the blood-brain barrier (BBB), 5-ALA can reach malignant cells, where it is metabolized into the fluorescent protoporphyrin IX (PpIX). Then, the fluorescent metabolite is thought to accumulate preferentially in tumor cells because of their decreased ferrochelatase levels compared to normal cells. Prospective data from a randomized Phase III clinical trial on 5-ALA FGS have demonstrated significant enhancements in progression-free survival (PFS) for patients with malignant glioma 8,9. Indeed, the group undergoing FGS experienced more complete tumor resection than patients following the standard treatment protocol, consistent with the improved overall survival. Nevertheless, 5-ALA can lead to false-positive fluorescence, for example in patients with recurring GBM, or false-negative fluorescence due to the diffuse nature of GBM 10. A recent study comparing FGS in 99 GBM cases with two optical imaging agents - 5-ALA and sodium fluorescein (SF), a less expensive compound not specific to GBM cells but able to identify BBB impairment - demonstrated their equal usefulness in achieving complete tumor resection 11. These results suggest that 5-ALA might not be the best option, leaving room for improvement. For example, to overcome some limitations of metabolic imaging, Rosenthal's group published the first-in-human FGS using tumor-targeting imaging 12,13. They reported the successful utilization of cetuximab, an antibody targeting EGFR (overexpressed in 50-70% of GBM) conjugated to the near-infrared fluorophore IRDye800CW to effectively resect GBM tumors under fluorescence, establishing the viability and the safety of this approach.

Another solution to improve GBM treatment involves the efficient selection of patients that are eligible for FGS. In this context, Li *et al.* reported the first-in-human study relying on a bimodal approach that enabled the preoperative assessment (*via* PET imaging) and FGS of GBM thanks to a bombesin peptide modified with both a ^68^Ga chelator and an IRDye800CW 14.

More recently, our group described the preclinical development of an anti-endothelin antibody and its Fab fragment to stratify patients suffering with GBM 15. Over the last three decades, extensive exploration of endothelin receptors and their role in cancer development has revealed a high expression on the surface of GBM cells and GBM stem cells, a subpopulation of highly tumorigenic cells responsible for tumor relapse 16-18. In particular, GlioVis Brain tumor transcriptomic dataset analysis from the TCGA GBM and the Ivy Glioblastoma Atlas Project clearly showed endothelin A receptor (ET_A_) overexpression in GBM patient biopsies, especially in the GBM vascular endothelium 19-21. Indeed, ET_A_ plays a critical role in regulating vascular tone and cell proliferation, making it a significant factor in tumor growth and progression. In addition, another study has shown that overexpression of ET_A_ is prevalent in a substantial portion of the glioblastoma multiforme patient population, contributing to the aggressive nature and poor prognosis of this malignancy 22. In our previous work, we demonstrated that the chimeric Rendomab A63 antibody (xiRA63) can be used in mice for the preclinical stratification of ET_A_^+^ glioblastoma by immunoPET imaging. Simultaneously, we developed a PET (^89^Zr-DFO)/OI (IRDye800CW) bimodal imaging probe (Monomolecular imaging platform MOMIP) that enabled the successful delineation of tumor margins - via fluorescence imaging - in mice bearing subcutaneous xenograft 23. Building upon these promising results, we report here the synthesis of a new version of the bimodal conjugate with a higher degree of labeling together with its preclinical evaluation in an orthotopic mouse model. This evaluation will explore its potential as i) a tool for the diagnosis and stratification of patients through PET imaging and ii) a support for visualizing tumor margins during surgery thanks to the fluorescence.

## Materials and Methods

### Antibody production

Production of xiRA63 antibody was previously described 15. In brief, heavy and light chains encoding xiRA63 were subcloned into the pTT5 expression plasmid 24. To remove the glycosylations carried by the antibody, the substitution of asparagine at position 297 with glutamine (N297Q) in the heavy chain was performed using the SLIC-PCR protocol on the pTT5 vector 25. The vectors were co-transfected into ExpiCHO-S cells (ThermoFisher Scientific) using the ExpiCHO Expression System Kit (ThermoFisher Scientific) following the manufacturer's instructions (MAN0014337 ThermoFisher Scientific). Subsequently, xiRA63-N297Q antibody - also called aglycosylated-xiRA63 (axiRA63) - was purified on a HiTrap Protein A HP column (GE HealthCare). Post-elution, antibody solutions were dialyzed with Slide-A-Lyzer™G2 Dialysis Cassette (ThermoFisher Scientific) into 1 L of phosphate-buffered saline (PBS).

### Preparation of the bimodal conjugate

MOMIP DFO-Tz-IR800 synthesis was previously described 23. Details can be found in the supplemental data.

### Enzymatic bioconjugation with MTGase providing the axiRA63-TCO

To a solution of axiRA63 was added TCO-PEG_4_-NH_2_ (60 equiv. at 60 mM in PBS), MTGase (5 U/mg of antibody) and phosphate buffered saline (PBS, pH 7.4) to adjust the antibody concentration to 7.5 g/L. The reaction was incubated at 25 °C for 2 h and then purified via size exclusion chromatography (Sephadex G-25 M, PD-10 column, GE Healthcare) and concentrated using centrifugal filter units with a 50,000 Da molecular weight cut off (Amicon^TM^ Ultra 2 mL Centrifugal Filtration Units, Millipore Corp., Billerica, MA) and phosphate buffered saline (PBS, pH 7.4). The degree of labeling was measured by mass spectrometry (DOL = 4) (Figure S1B).

### axiRA63-MOMIP by Inverse electron demand Diels Alder reaction

To a solution of axiRA63-TCO was added the MOMIP DFO-Tz-IR800 (10 equiv. *per* TCO at 30 mM in DMSO) and the antibody concentration was adjusted to 5 g/L by adding phosphate buffered saline (PBS, pH 7.4). The reaction mixture was stirred at room temperature overnight and purified by Protein A affinity chromatography (Hitrap Mabselect Xtra column, Cytiva) to give axiRA63-MOMIP. The degree of labeling was measured by UV spectroscopy (DOL = 3.1 ± 0.1) and mass spectrometry (DOL = 2.5) (Figure S1C).

### Flow cytometry

We used the same protocol as previously detailed in Herbet *et al.* 26. To evaluate and compare the apparent affinity of axiRA63 and axiRA63-MOMIP, two cell lines were used: Chinese hamster ovary wild-type (CHO-WT) cells and CHO cells stably transfected with human ET_A_ (CHO-ET_A_). Antibody dilutions ranging from 0.001 nM to 10 nM were performed to estimate the apparent affinity. Alexa-Fluor 488^TM^ conjugated with F(ab')2-goat anti-human IgG Fc (Invitrogen) was incubated for 4 h at 4 °C. Following each step, samples underwent three washes with PBS. Fluorescence intensity at 488 nm was measured using the FACSCalibur flow cytometer (BD Biosciences) and expressed as a percentage of the mean fluorescence intensity per sample. The “one site specific binding” function of GraphPad was applied to fit the binding curve, and determine the Bmax and the apparent Kd values (Figure S2).

### Cell culture Gli7

A previously described human GBM stem cell line called Gli7 27 which express ET_A_^+^ was used and cultured in DMEM-F12 (Gibco) supplemented with 1% glutamine (Gibco), 1% B27 (Gibco), 1% N2 (Gibco), 20 µg/mL insulin (Sigma), 0.6 µg/mL glucose (Sigma), 0.2 µg/mL EGF (Prepotech), 0.2 µg/mL bFGF (Preprotech), 2 µg/mL heparin (Sigma) and 2 µg/mL ciprofloxacin (Euromedex). Maintenance of this cell line was performed at 37 °C in a 5% humidified atmosphere.

### Mouse model orthotopically implanted

NMRI nude mice (Janvier Labs, female, 4 weeks old) were orthotopically implanted with 2 µL containing 5.10^5^ Gli7 cells (n = 8) or with PBS (n = 3), into the striatum (2 mm on the right from the bregma and 2.5 mm depth from the dura). Animal anesthesia was induced using 2.5% isoflurane (4% for induction) in oxygen, coupled with local xylocaine anesthesia. Buprenorphine at dose 0.05 mg/kg was administered subcutaneously during surgery. All animal experiments adhered to the guidelines set by the European Directive 2010/63/EU and to its transposition into the French law (Decree No. 2013-118).

### Immunoconjugates radiolabeling with ^89^Zr

Immunoconjugates were radiolabeled with Zirconium-89 (PerkinElmer) following a well-established methodology 28. Briefly, on the day preceding injection, 1.2 mg of axiRA63-MOMIP, pH adjusted to 7.2, were incubated for 49 min at 37 °C, 300 rpm in a solution containing neutralized [^89^Zr][Zr-(oxalate)_4_]^4-^ with Na_2_CO_3_ (2 M). Purification of the [^89^Zr]Zr-axiRA63-MOMIP was performed on a PD-10 column with a gentisic acid solution (pH 5.4 - 5.6) as the mobile phase and concentrated with Vivaspin® ultrafiltration tubes (Sartorius, cutoff: 50 kDa). Radiochemical purity of [^89^Zr]Zr-axiRA63-MOMIP was estimated using instant thin layer chromatography (iTLC-SG glass microfiber papers impregnated with silica gel (Agilent Technologies)). The mobile phase comprised a citric acid solution with 5% acetonitrile (20 mM, pH 4.9 - 5.1) for the elution. iTLC results were read with a Mini-Scan TLC Imaging Scanner (Eckert & Ziegler). Radiochemical purity of [^89^Zr]Zr-axiRA63-MOMIP was confirmed by size-exclusion high-performance liquid chromatography (HPLC) coupled with a UV (UVD 170U UV/VIS) and scintillation gamma detector (Packard). Measurements were conducted using a bioZen 1.8 µm SEC-2 LC column (Phenomenex) and a DIONEX System (Thermo-Fisher). A linear-gradient elution was performed with a solution of KH_2_PO_4_ (50 mM) and KCl (250 mM) (pH 6.8), at a flow rate of 0.2 mL/min (Figure S3). Radiolabeled immunoconjugate was subsequently diluted with cold antibody to reduce the total sample radioactivity.

### Antibody injection

On day 79 post-implantation of GBM cells, according to the tumor development progression outlined in a previous study 15, 8 tumor bearing mice were randomized into two groups (Figure S4): the first group (n = 5 mice) received intravenous administration of [^89^Zr]Zr-axiRA63-MOMIP (4.25 MBq ± 0.10 MBq; 82.7 µg ± 1.9 µg) whereas the second (n = 3 mice) was pre-injected with murine RA63 (1 mg in 100 µL) 72 h prior to the injection of the [^89^Zr]Zr-axiRA63-MOMIP (4.25 MBq ± 0.10 MBq; 82.7 µg ± 1.9 µg ). The controlled group implanted with PBS (n = 3 mice) received intravenous administration of [^89^Zr]Zr-axiRA63-MOMIP (4.25 MBq ± 0.10 MBq; 82.7 µg ± 1.9 µg). Specific activities injected are detailed in supplementary Table S1.

### PET-CT image acquisition

PET acquisitions were conducted using the Inveon microPET-CT system (Siemens Medical Solutions, Knoxville, TN, USA) providing a spatial resolution of approximately 1.5 mm (FWHM). Following the PET scan, a 6-minute 80 kV/500 µA CT scan was performed for attenuation correction. PET images were reconstructed using a 3D OSEM iterative algorithm with parameters set to 4 iterations, 16 subsets, and a voxel size of 0.4 mm × 0.4 mm × 0.8 mm. The reconstruction process included normalization, dead time correction, random subtraction, CT-based attenuation correction, and scatter correction. A 20-minute PET acquisition was performed at 48 h [^89^Zr]Zr-axiRA63-MOMIP post injection (p.i.).

### PET-CT analysis

The analysis of PET-CT data was conducted using PMOD software (Version 3.9). For brain tumor assessment, volumes of interest (VOI) were delineated using isocontour methodology. The same tumor's VOI was applied to the contralateral side of the brain (used as a negative control). The activity concentration in each VOI was corrected based on the half-life of ^89^Zr and presented as a percentage of injected dose per volume of tissue (%ID/cm^3^).

### MRI

On day 61 post Gli7 implantation, anatomical T1-weighted contrast-enhanced MRI scans were obtained using a 7T/90 mm bore hole MRI scanner (Pharmascan scanner, Bruker) following intravenous injection of Gadolinium-based contrast agent (Dotarem®, 1nm diameter, 100 µL by animal). The following parameters were used: MSME sequence, TE/TR = 8/340 ms, matrix = 256 × 256 × 64, resolution = 0.15 × 0.15 × 0.60 mm^3^, 10 averages, acquisitions time = 6 min.

### PET and MRI image fusion analysis

PET and MRI transverse sections images were fused using PMOD software (Version 3.9) and were all aligned via the T1w MRI as the reference for all analyses, with the rigid transformation.

### Assessment of the [^89^Zr]Zr-axiRA63-MOMIP antibody stability by iTLC

The *in vivo* [^89^Zr]Zr-axiRA63-MOMIP stability was assessed at 48 h p.i. by iTLC. Details can be found in the supplemental data (Figure S6).

### KIS800 Fluorescent Imaging System (Kaer Labs)

The KIS 800 imaging system (Kaer Labs) was employed for real-time fluorescence imaging of brain tumors, enabling simultaneous surgery and fluorescence imaging. The system includes a laser with an excitation wavelength of 785 nm and a collection filter to detect fluorescence emitted above 810 nm. During the acquisition, three raw images are acquired at each time point through Kaer Labs' proprietary software: a fluorescent image obtained by exciting the fluorophore with the 785 nm laser and collecting emission above 810 nm, a background noise image representing emissions at more than 810 nm without prior excitation, and a "bright" image obtained with an infrared LED to detect the animal. From these three images, a corrected image - corresponding to the subtraction of background signal from the fluorescent image - is generated in real time. In addition, an overlay image can be generated to visualize the latter superimposed onto the bright image.

The same excitation time was applied across different groups of mice. A 500 ms exposure time was used when comparing the different groups on the whole mice, while a 100 ms time of exposure was enough to compare brain tumor with and without the skull. Fluorescence-guided tumor resection was performed using an exposure time of 100 ms. This short exposure time enabled the acquisition of videos in ".tiff" format. Finally, one of the resected brains with residual fluorescent signal was also imaged at exposure time of 100 ms and 200 ms.

### *Ex vivo* analysis

Following resection of the tumor, one of the brains with residual fluorescence was frozen in liquid nitrogen and stored at -80 °C. Coronal brain sections were cryo-sectioned into 12 µm thick slices using a cryostat (Leica CM3050 S, Leica biosystems). Some slices were stained with Harris hematoxylin and Eosin (Sigma-Aldrich) to confirm the presence of any residual tumor. The sections were fixed with 10% neutral buffered formalin, incubated 2 min in Harris hematoxylin (Sigma-Aldrich) and rehydrated in a 70% ethanol solution with 0.25% hydrochloric acid (HCl). Subsequently, sections were colored with Scott substitute (2 g NaHCO_3_, 20 g MgSO_4_) for 1 min. Slides were incubated 30 s in 50% ethanol solution, followed by 70% ethanol solution and 1.5 min in Eosin Y alcoholic (Sigma-Aldrich). Sections were left three times during 3 min in 95% ethanol solution, then 100% ethanol solution and in toluene (VWR Chemicals). Finally, sections were mounted using Eukitt Quick-hardening mounting medium (Fluka Analytical). Axio Observer Z1 microscope (Zeiss, Germany) with a 20x objective was used to scan the full sections. Post-processing qualitative analyses were carried out with ZEN software (v2.6, Zeiss).

One of the Gli7 tumor brain injected with [^89^Zr]Zr-axiRA63-MOMIP, was frozen at -80 °C at 48 h p.i. without the tumor being resected. The whole frozen brain was cryosectioned into 12 μm thick sections, and the resulting tumor sections were imaged with the Kratoscope imaging system (Kaer Labs) allowing the 3D reconstruction of the fluorescence. The surface of the block was illuminated with a 785 nm laser source to detect dual labeled antibody fluorescence, and infrared (IR) LED were used to generate a brightfield image of the organ. To demonstrate co-localization between fluorescence and radioactivity, individual sections were collected and [^89^Zr]Zr-axiRA63-MOMIP distribution was assessed by autoradiography using a phosphor-screen and read with a phospho-imager (a Storm 860 Molecular Imager, Molecular Dynamics). The signal emitted by the screen corresponds directly to the radioactivity on the slice.

### Statistics

Statistical analyses were performed using GraphPad Prism software (v9.0.1). Student t-tests (two-tailed) were conducted to compare two data groups (* P < 0.05, ** P < 0.01, *** P < 0.001, **** P < 0.0001).

## Results

### Preparation and* in vitro* characterization of axiRA63-MOMIP

**Figure 1:**
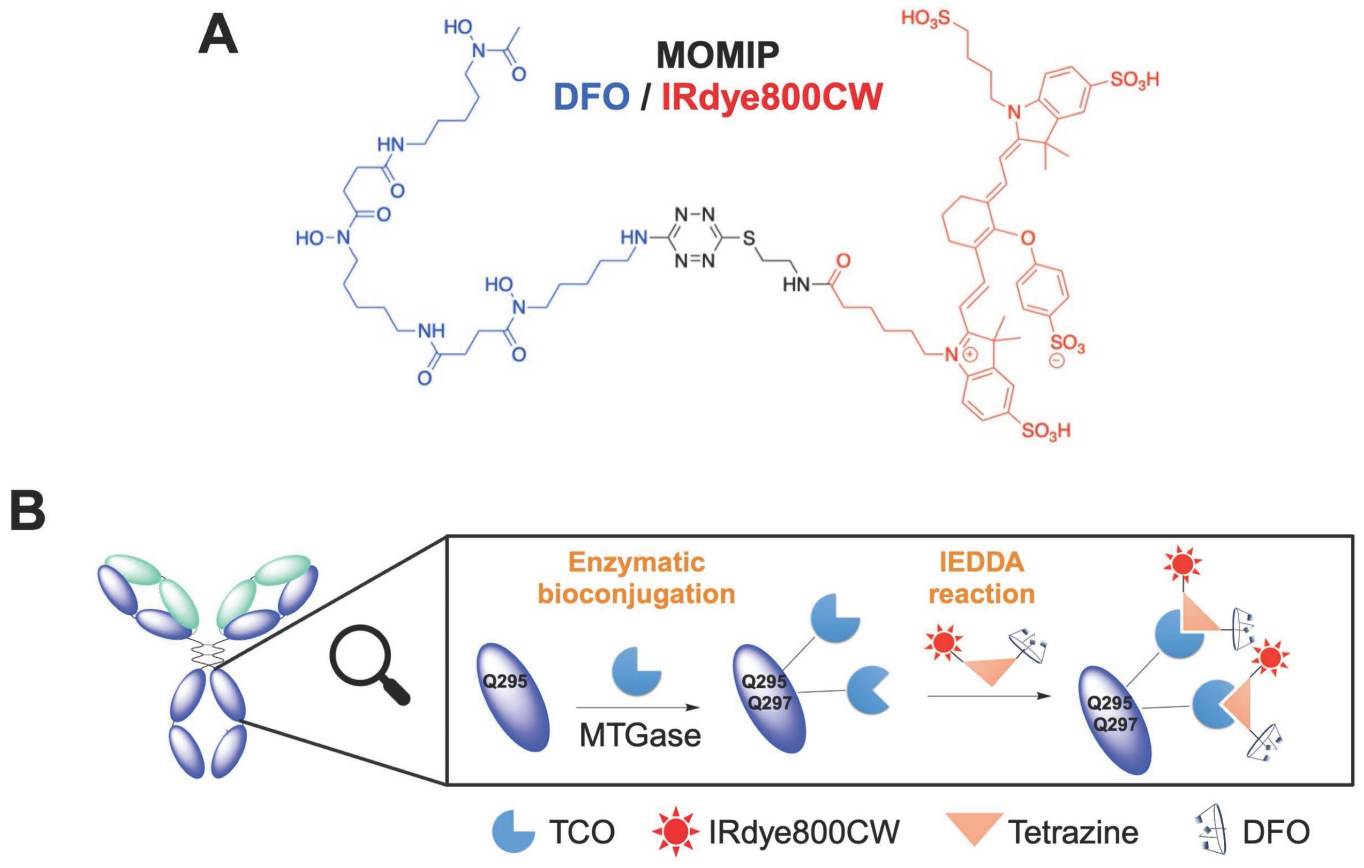
** Synthesis of the bimodal imaging agent. A.** Structure of the MOMIP.** B.** Two-step process bioconjugation to the antibody: 1) Enzymatic conjugation with MTGase to introduce the TCO moiety; 2) addition of the MOMIP *via* an Inverse Electron Demand Diels-Alder (IEDDA) reaction.

The MOMIP synthesis details and the conjugation method have been previously described by our group 23. To maximize the degree of labeling (DOL: number of MOMIP per antibody), an aglycosylated version of the chimeric antibody (axiRA63), containing four free glutamines, was engineered. Then, a two-step process - i) introduction of a TCO moiety through enzymatic conjugation with transglutaminase; ii) addition of the MOMIP *via* an Inverse Electron Demand Diels-Alder (IEDDA) reaction (Figure 1, Figure S1A) - was used to obtain the bimodal conjugate axiRA63-MOMIP, with a DOL between 2.5 and 3.1 ± 0.1 as measured by mass spectrometry and UV analysis. (Figure S1C). Thanks to our previous work, we know that there is no other reactive glutamine on the light or heavy chains 23.

To confirm that the site-specific conjugation did not impair the binding ability, we compared the functionality of the native antibody axiRA63 with the axiRA63-MOMIP conjugate by flow cytometry (Figure S2). Antibodies specificity was maintained as the CHO-WT negative control cells generated signals below 1% MFI (Median Fluorescence Intensity) whereas a high robust signal was obtained on CHO-ET_A_^+^ cells. Besides, axiRA63-MOMIP retained a high apparent affinity in the nanomolar range (0.05 nM ± 0.001 nM vs 0.28 nM ± 0.002 nM for the native antibody). However, the Bmax of the bimodal conjugate (36.95 %MFI ± 0.06 %MFI) was significantly lower than the native antibody one (94.37 %MFI ± 0.12 %MFI). This result may be attributed to steric hindrance arising from the addition of multiple MOMIPs on the Fc region of the antibody, resulting in loss of axiRA63-MOMIP recognition by the secondary antibody.

### Preoperative PET imaging and biodistribution: [^89^Zr]Zr-axiRA63-MOMIP as a diagnosis tool

axiRA63-MOMIP was subsequently radiolabeled with ^89^Zr using standard protocols, generating the radioimmunoconjugate [^89^Zr]Zr-xiRA63-MOMIP in 95% radiochemical yield and 100% radiochemical purity (Figure S3).

To evaluate the *in vivo* behavior of our radioimmunoconjugate in a clinically relevant model, we orthotopically implanted Gli7 ET_A_^+^ GBM stem cells into nude mice brains (8 mice). In the meantime, a control group (3 mice) received a stereotaxic injection of PBS (instead of tumor cells). At 61 days post-implantation, mice were imaged by contrast MRI to visualize BBB impairment and confirm tumor development. Gli7 tumor-bearing mice were randomly divided in two groups: the “Surgery group” (5 mice) dedicated to fluorescence-guided surgery of glioblastoma, and the “Blocking group” (3 mice). The latter was intravenously pre-injected with 1 mg of murine Rendomab A63 (RA63), a non-radioactive competitor blocking ET_A_ binding sites (Figure S4), on day 76. On day 79 post implantation, all mice (control, surgery and blocking group) were injected with [^89^Zr]Zr-axiRA63-MOMIP (4.25 MBq ± 0.10 MBq; 82.7 µg ± 1.9 µg) (Table S1) and whole-body imaged by microPET/CT at 48 h p.i. Despite a significant delay between the two imaging techniques - allowing further tumor growth and BBB alteration - we were able to verify the co-localization of PET signal and tumor. The biodistribution profile of [^89^Zr]Zr-axiRA63-MOMIP was consistent with its non-fluorescent version reported previously in Hautiere *et al.* 2023 15. Briefly, [^89^Zr]Zr-axiRA63-MOMIP was excreted by the hepato-biliary system, with prominent accumulation in the liver at 48 h p.i. due to its high molecular weight (>60 kDa) (Figure S5). *In vivo* plasmatic antibody stability, assessed at 48 h post-injection using iTLC, confirmed that there is no free zirconium in the blood.

**Figure 2:**
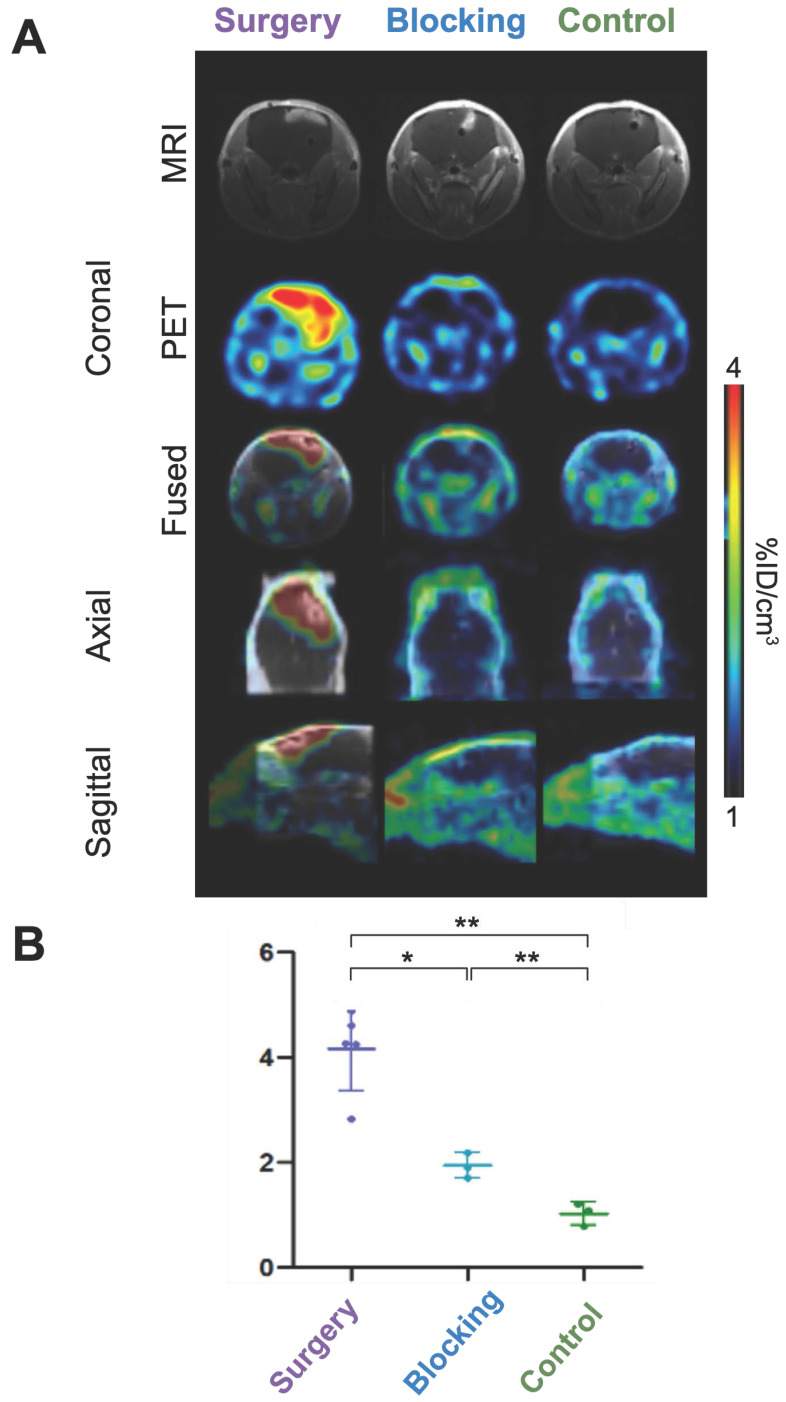
** Brain tumor uptake and immunoPET imaging of [^89^Zr]Zr-axiRA63-MOMIP at 48 h p.i. A.** PET and MRI images. Brain tumor coronal section by MRI with contrast agent obtained 18 days before injection and by PET imaging at 48 h p.i. for the three groups: surgery, blocking and control. **B.** Quantitative uptake of [^89^Zr]Zr-axiRA63-MOMIP in the Gli7 model. Data are presented as mean ± SD; statistical comparisons were performed using a two-tailed paired Student's t test. **P < 0.01; *P < 0.05; ns = not significant; n (mice surgery group) = 5; n (mice blocking group) = 3; n (mice control group) = 3.

To assess the diagnostic potential of our conjugate, brain tumor uptake was compared between the different groups. In the surgery group, tumors were clearly delineated, while little to no signal was observed in the control and blocking groups, respectively (Figures 2A-B, Figures S7-8). Quantitative tumor uptake extrapolated from PET imaging confirmed these results, with an average [^89^Zr]Zr-axiRA63-MOMIP accumulation of 4.2 %ID/cm^3^ ± 0.8 %ID/cm^3^ in the surgery group *vs* 1.0 %ID/cm^3^ ± 0.2 %ID/cm^3^ (** p < 0.01 compared to the surgery group) in the control group and 1.9 %ID/cm^3^ ± 0.2 %ID/cm^3^ (* p < 0.05) in the blocking group (Figure 2B). The absence of [^89^Zr]Zr-axiRA63-MOMIP uptake in the control group demonstrated that the stereotaxic implantation does not create false positive signal.

### *In vivo* assessment of brain tumor fluorescence imaging mediated by [^89^Zr]Zr-axiRA63-MOMIP and correlation with immunoPET imaging

**Figure 3:**
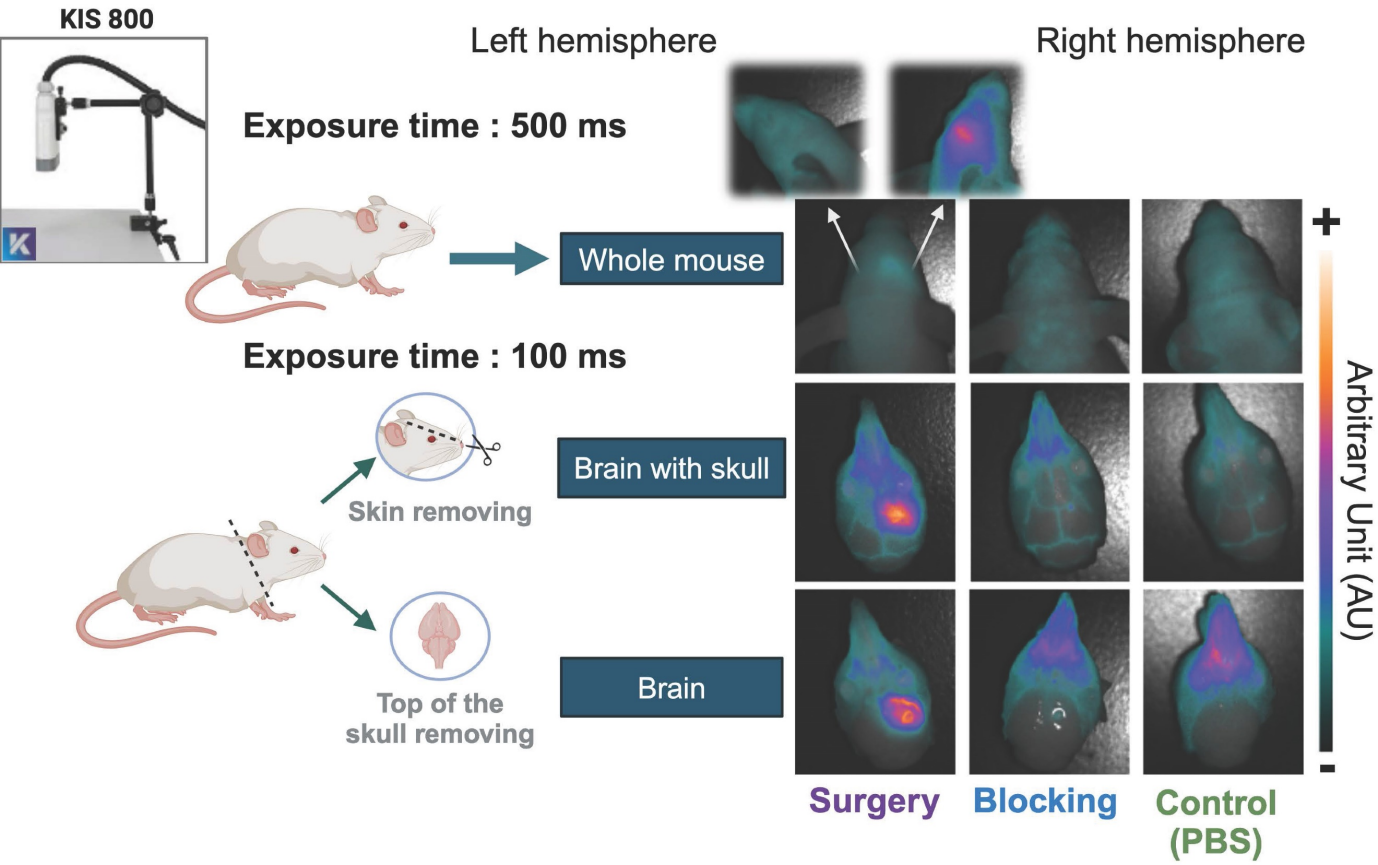
** Post mortem fluorescence images during different dissection stages.** Fluorescent images were acquired post mortem at 48 h p.i. of [^89^Zr]Zr-axiRA63-MOMIP-IR800, with the KIS 800 imaging system (Kaer Labs). The first set, called "whole mouse”, includes an image of the mouse head with skin, skull and brain with an exposure time of 500 ms. Next, an image of the mouse head skull and brain, called "brain with skull", was taken on all three groups of mice, after skin removal. Finally, the last set of images, called "brain", was taken after removal of the upper cranial cavity. The last two conditions required an exposure time of 100 ms.

Before performing the FGS on the surgery group, we assessed the fluorescence of the dual-labeled antibody [^89^Zr]Zr-axiRA63-MOMIP in the three groups of mice using the KIS 800 imaging system (Kaer Labs). The 48h p.i. imaging timepoint was chosen to obtain the highest fluorescence signal, according to our last study 23. Figure 3 illustrates the results of fluorescent images obtained at different steps, from the whole mouse up to the fully exposed brain. As anticipated, no observable fluorescence signal was detected at the cerebral cortex level in the "control (PBS)" or "blocking" groups, regardless of whether the skin or skull was removed. It should be noted that the fluorescent signal in the blocking and control groups appears at the snout due to bone structure and light reflection. However, in the surgery group, a distinct fluorescence signal corresponding to the tumoral uptake of [^89^Zr]Zr-axiRA63-MOMIP was evident even without removing the skin. Upon removal of the skin and skull, the tumor border can be clearly delineated. To evaluate the specificity of the fluorescence signal, an image was captured displaying the whole brain hemispheres with the tumor ipsilateral side (right hemisphere) and the corresponding healthy contralateral side (left hemisphere). No signal was observed on the contralateral side, confirming the specificity of the fluorescence signal to the tumor.

**Figure 4:**
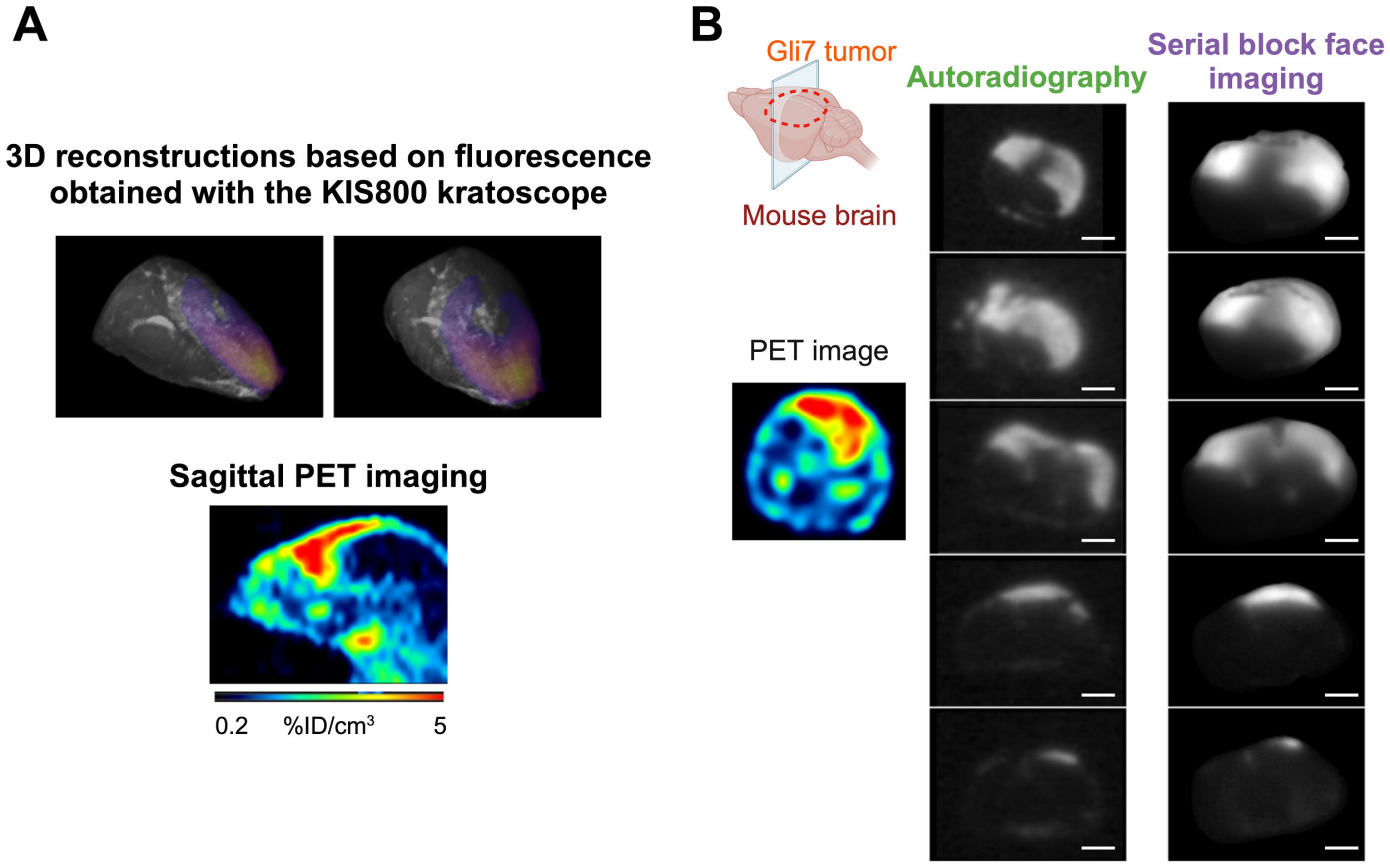
***Ex vivo* co-localization of the fluorescent and nuclear signals in the brain tumor 48h p.i. of [^89^Zr]Zr-axiRA63-MOMIP. A.** Comparison of the 3D fluorescence reconstruction using the kratoscope (Kaer Labs) and the sagittal PET nuclear imaging. **B.** Comparison of nuclear and fluorescent signal on corresponding brain tumor sections obtained by autoradiography and block face fluorescence imaging (scale bars represent 2.4 mm).

To enhance the efficacy of Fluorescence-Guided Surgery (FGS), achieving a precise co-localization of PET and fluorescence signals is imperative. Therefore, an *ex vivo* analysis was performed to confirm the correlation between the two different signals. For this purpose, one tumor bearing brain from the surgery group was collected, immediately frozen, and completely sectioned. Serial block face imaging of the sample was performed using the Kratoscope system (Kaer Labs, France) to detect tissue fluorescence of the harvested organ. Briefly, this method generates serial images of the block's surface, generating a stack of 2D images which can then be registered and reconstructed to obtain the 3D volume of the sectioned organ. The block was imaged using 785 nm excitation laser and 850 nm IR LED to respectively detect labeled-antibody specific fluorescence and unlabeled tissue, and a 3D reconstruction of the entire brain's fluorescent signal was generated (Figure 4A). Comparison of the signals obtained from immunoPET (sagittal axis) and fluorescence 3D reconstruction across the entire brain revealed a consistent geometry and profile between the two modalities. To definitively correlate these PET and fluorescent signals, 2D sections collected during the serial sectioning and imaging with the Kratoscope - spread uniformly over the entire tumor mass to ensure the homogeneity of the response across the tumor - were also subjected to autoradiography. As illustrated in Figure 4B, the signal obtained through autoradiography precisely mirrored the fluorescence signal. This experiment demonstrated the perfect co-localization of the fluorescent and radioactive signals and validated the potential of the [^89^Zr]Zr-axiRA63-MOMIP antibody as a theranostic tool for the diagnostic and FGS of glioblastoma.

### [^89^Zr]Zr-axiRA63-MOMIP, a gold candidate for the fluorescence guided surgery of ET_A_^+^ GBM

**Figure 5:**
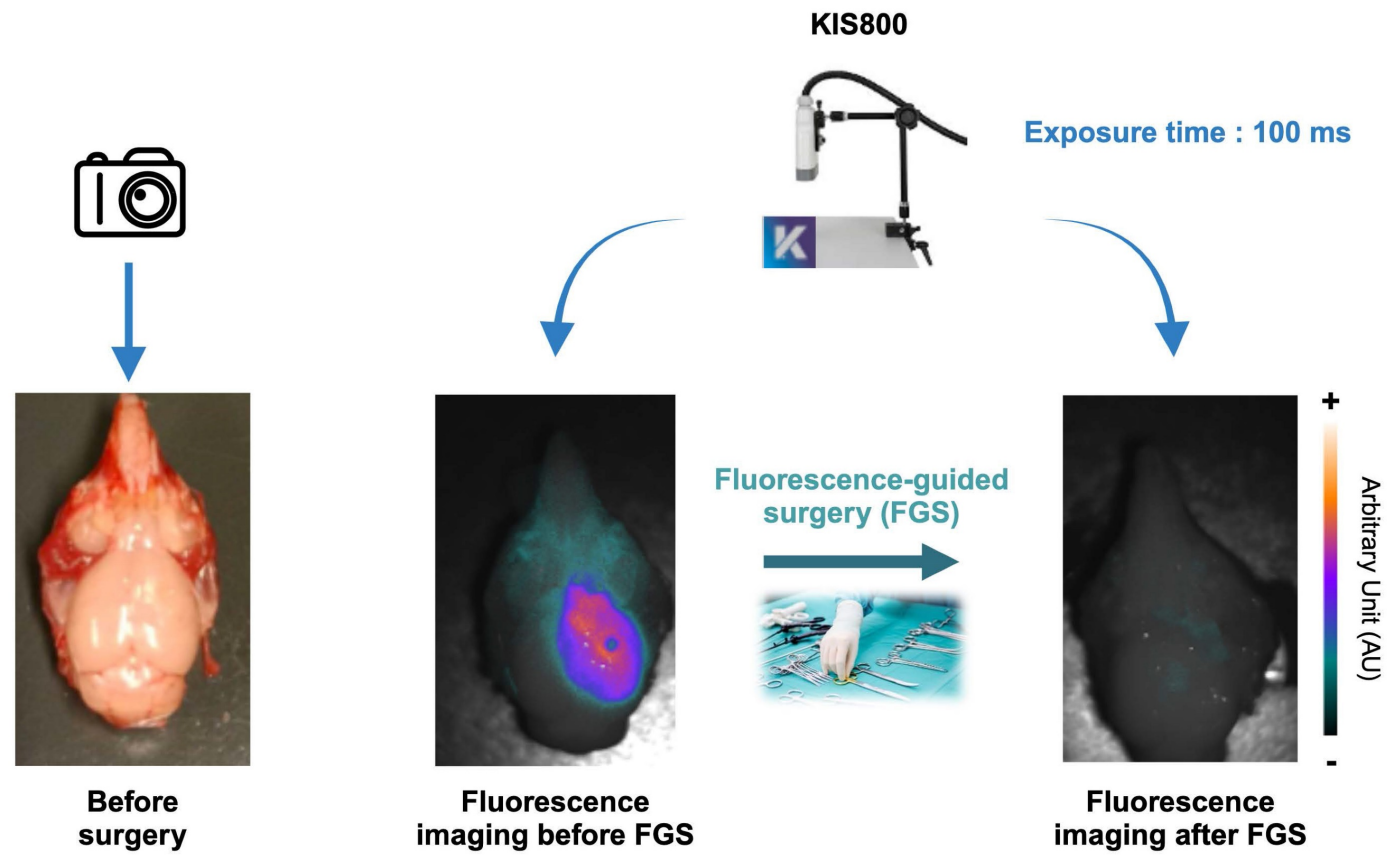
** Example of an e*x vivo* FGS performed post mortem on the GBM preclinical model:** Left image shows the brain tumor Gli7 ET_A_^+^ before surgery without fluorescence signal. Middle image corresponds to the brain Gli7 ET_A_^+^ tumor viewed by fluorescence with a time exposure of 100 ms. Right image shows an image of a resected brain guided with fluorescence *via* the KIS 800 system (Kaer Labs) with a time exposure of 100 ms.

The feasibility of Fluorescence-Guided Surgery (FGS) using [^89^Zr]Zr-axiRA63-MOMIP was substantiated post-mortem, 48 h after injection. Live FGS using KIS800 camera is available in the supplemental data. As shown in Figure 5 and Figure S9, the preoperative fluorescence signal exhibited remarkable intensity in all subjects within the surgery group (except for the mouse without MRI signal), allowing a clear delineation of tumors. In contrast, a picture taken under white light highlights the visual challenges faced by neurosurgeons to distinguish healthy tissue from tumor tissue. During the surgical procedure, fluorescence was a valuable aid to identify malignant cells.

### Post-resection observation of weak residual fluorescence signal validates the relevance of using [^89^Zr]Zr-axiRA63-MOMIP to achieve complete tumor removal

**Figure 6:**
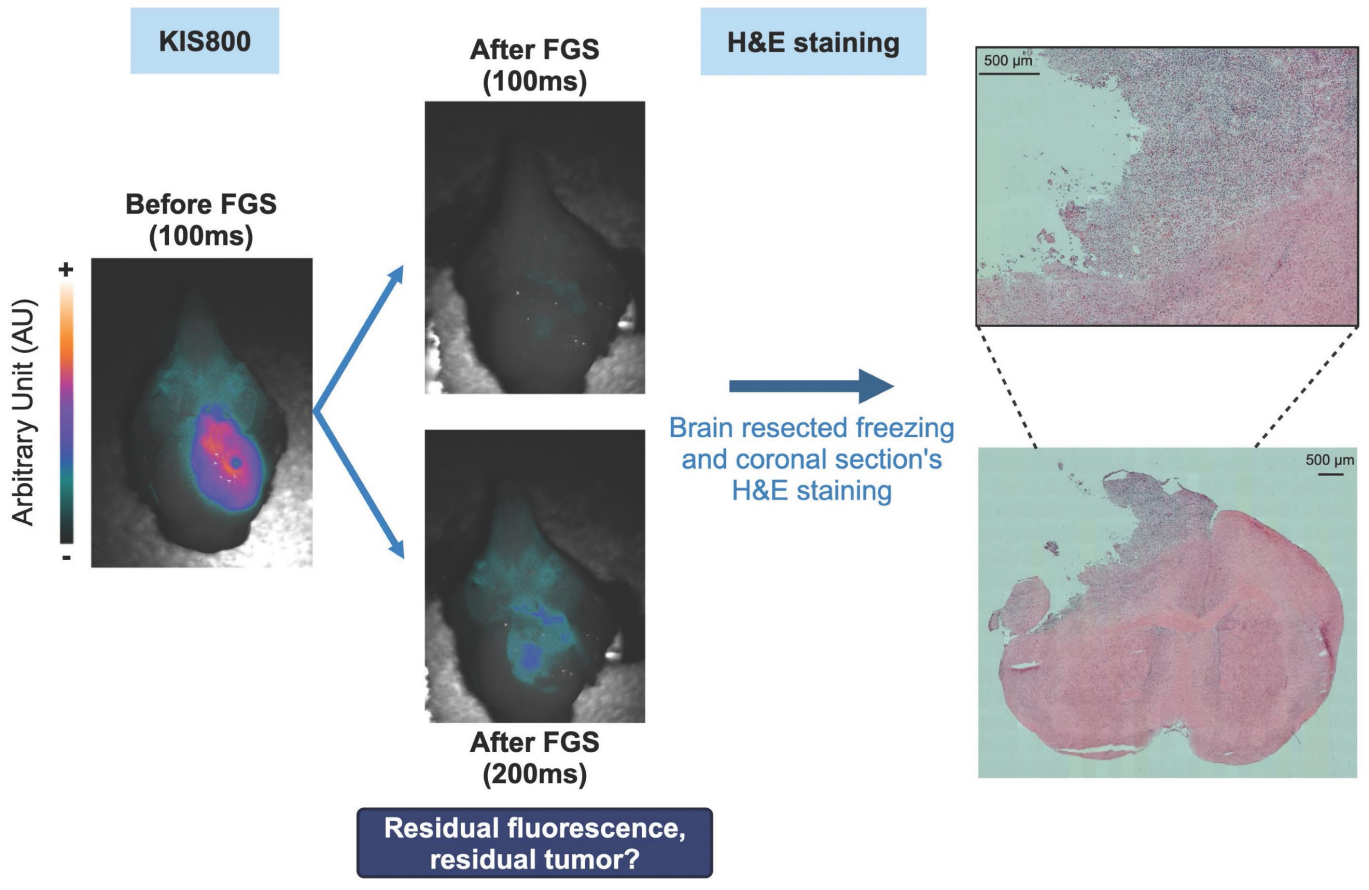
** E*x vivo* assessment of the GBM's fluorescence-guided surgery quality:** Fluorescent tumor residues imaged by the KIS 800 at 100 ms, 200 ms and H&E coloration of brain sections corresponding to these residues.

Following the resection, a residual weak fluorescence signal was detected with a prolonged exposure time (200 ms), suggesting a near-complete removal of the tumor mass (Figure 6). To verify whether the presence of this residual fluorescence was due to the existence of tumor cells infiltrating the healthy tissue or to a non-specific signal, one resected brain was frozen, cut in several coronal sections which were H&E stained. Coloration results are presented in Figure 6 and confirmed the persistence of tumor cells after resection which were well correlated with the fluorescence signal.

## Discussion

Achieving maximal surgical resection remains the best treatment option to extend the survival of GBM patients. However, the challenges posed by the invasive nature of the disease, especially in the eloquent brain regions, are narrowing the chances of successful surgery 6. Indeed, surgeons often face the dilemma of removing malignant tissue without compromising healthy regions. In the last decades, Fluorescence-Guided Surgery, primarily utilizing 5-ALA, has gained popularity due to its cost-effectiveness, simplicity, and safety. Despite these advantages, 5-ALA presents limitations, including low sensitivity for low-grade lesions (glioma I - III or recurrent GBM with lower-grade regions), potential false positives in radiation necrosis regions, and the absence of non-invasive stratification technique to select eligible patient for a successful FGS.

Therefore, there is a need for a tool that will help stratify GBM patients. While MRI with a contrast agent remains a primary diagnostic option, it cannot distinguish radionecrosis regions from tumor relapsing 29. Recently, amino acid PET imaging, recommended by the Response Assessment in Neuro-Oncology (RANO) working group, has emerged as an additional brain tumors diagnostic tool 30. Multimodal imaging - combining a PET radioisotope for diagnosis/stratification and a fluorophore for tumor delineation on a single agent - represents an enticing solution to treat GBM. A few studies have reported this strategy, using different carriers: peptides 14,31, nanoprobes 32,33 or protein complex 34*.* In the present work, we developed the first antibody-based bimodal imaging agent, specifically targeting ET_A_ receptors overexpressed in GBM stem cells and on the vascular endothelium close to the GBM 19,21. This probe demonstrated high selectivity and active tumor targeting, overcoming some of 5-ALA limitations. Besides, due to the low expression of ET_A_ receptors in healthy brain, the occurrence of false positive signal is unlikely 35.

A key point to properly assess the efficacy of our probe was to find a clinically relevant model. Whereas most of the previously cited bimodal imaging studies used U87MG human glioma cells, we opted for an orthotopic implantation of patient-derived Gli7 cells providing a model that closely mimics the development of GBM in a patient. Indeed, animals do not exhibit clinical signs for several weeks, highlighting the insidious invasiveness of this preclinical model, which is similar to clinical observations 27. Besides, it is important to note that, like most of the cited literature, we rely on a disrupted blood-brain barrier, also called blood-tumor barrier, to reach the tumor. Indeed, crossing the BBB is essential to reach the tumor, yet it poses a significant challenge due to antibody size. While the alteration of the BBB facilitating antibody penetration is well-documented in advanced tumors 36, it may remain intact in emerging tumors or migrating tumor cells 37. In those cases, strategies to open the BBB, like therapeutic ultrasounds 38, are therefore needed and we are currently exploring different ways to overcome this limitation. In our study, all animals except one exhibited a disrupted BBB - as evidenced by the passage of DOTAREM, an MRI contrast agent that typically does not cross the BBB in its intact state - which is representative of the disease condition. Consistent with our previous study, PET imaging showed a highly specific signal in tumors from the surgery group 15. The enhanced passage of the full-sized antibody [^89^Zr]Zr-xiRA63 may be facilitated by the increased permeability of this model compared to others, or more likely, through alternative mechanisms such as the expression of ET_A_ on endothelial cells 39. Foreseeably, PET imaging of the sole mouse presenting no visible BBB impairment revealed a lower tumor accumulation of our tracer compared to other mice presenting contrast enhanced MRI (Figures S7-8). This result highlights the critical role of the BBB, regardless of ET_A_ expression. The residual signal observed in the "blocking" group, despite of rendomab A63 occupancy, can be explained by the dynamics of G protein-coupled receptors (GPCRs) and their continuous renewal at the tumor membrane level 40. Fluorescence imaging enabled an accurate delineation of tumor margins, even before skull removal. This can be attributed to the near-infrared emission of IRDye800CW, allowing deeper tissue penetration and representing a tremendous advantage over 5-ALA to identify tumor cells deeply infiltrated in the brain. Moreover, since the excitation and emission wavelengths of IRDye800CW overlap with those of indocyanine green (ICG) - another fluorophore used to perform FGS in other types of cancer - there is no need for hospitals to invest in new materials or training. Remarkably, qualitative correlation between PET and fluorescence signals is a favorable outcome as it suggests that an effective patient selection would be achievable.

In conclusion, the dual-labeled antibody [^89^Zr]Zr-axiRA63-MOMIP has successfully enabled both pre-operative PET imaging and fluorescence-guided resection of a human GBM in a preclinical rodent model. Unlike most of the fluorophores already in the clinic, our bimodal probe, conjugated to a highly specific antibody, could represent a much-needed tool to stratify patient eligible for a successful FGS. However, in patients with early-stage GBM, where the BBB is most likely intact, our dual-labeled antibody may be less effective than in the preclinical models studied herein. Moving forward, efforts will focus on improving BBB penetration to explore the development of a novel therapeutic treatment.

## Supplementary Material

Supplementary materials and methods, figures, table, and video legends.

Supplementary video 1.

Supplementary video 2.

Supplementary video 3.

## References

[1] Louis DN, Perry A, Wesseling P, Brat DJ, Cree IA, Figarella-Branger D (2021). The 2021 WHO classification of tumors of the central nervous system: a summary. Neuro Oncol.

[2] Rodríguez-Camacho A, Flores-Vázquez JG, Moscardini-Martelli J, Torres-Ríos JA, Olmos-Guzmán A, Ortiz-Arce CS (2022). Glioblastoma treatment: state-of-the-art and future perspectives. Int J Mol Sci.

[3] Brown TJ, Brennan MC, Li M, Church EW, Brandmeir NJ, Rakszawski KL (2016). Association of the extent of resection with survival in glioblastoma. JAMA Oncol.

[4] Wykes V, Zisakis A, Irimia M, Ughratdar I, Sawlani V, Watts C (2021). Importance and evidence of extent of resection in glioblastoma. J Neurol Surg A Cent Eur Neurosurg.

[5] Fernandes C, Costa A, Osório L, Lago RC, Linhares P, Carvalho B (2017). Current standards of care in glioblastoma therapy. In: De Vleeschouwer S, Ed. Glioblastoma. Brisbane (AU): Codon Publications.

[6] Gerritsen JKW, Broekman MLD, De Vleeschouwer S, Schucht P, Nahed BV, Berger MS (2022). Safe surgery for glioblastoma: Recent advances and modern challenges. Neuro-Oncol Pract.

[7] Hadjipanayis CG, Stummer W (2019). 5-ALA and FDA approval for glioma surgery. J Neurooncol.

[8] medac GmbH (2012). Fluorescence-guided resection of malignant gliomas with 5-Aminolevulinic acid (5-ALA) vs. conventional resection [Internet].

[9] Stummer W, Pichlmeier U, Meinel T, Wiestler OD, Zanella F, Reulen H-J (2006). Fluorescence-guided surgery with 5-aminolevulinic acid for resection of malignant glioma: a randomised controlled multicentre phase III trial. Lancet Oncol.

[10] Hadjipanayis CG, Widhalm G, Stummer W (2015). What is the surgical benefit of utilizing 5-ALA for fluorescence-guided surgery of malignant gliomas?. Neurosurgery.

[11] Zeppa P, De Marco R, Monticelli M, Massara A, Bianconi A, Di Perna G (2022). Fluorescence-guided surgery in glioblastoma: 5-ALA, SF or both? Differences between fluorescent dyes in 99 consecutive cases. Brain Sci.

[12] Warram JM, de Boer E, Korb M, Hartman Y, Kovar J, Markert JM (2015). Fluorescence-guided resection of experimental malignant glioma using cetuximab-IRDye 800CW. Br J Neurosurg.

[13] Miller SE, Tummers WS, Teraphongphom N, van den Berg NS, Hasan A, Ertsey RD (2018). First-in-human intraoperative near-infrared fluorescence imaging of glioblastoma using cetuximab-IRDye800. J Neurooncol.

[14] Li D, Zhang J, Chi C, Xiao X, Wang J, Lang L (2018). First-in-human study of PET and optical dual-modality image-guided surgery in glioblastoma using ^68^Ga-IRDye800CW-BBN. Theranostics.

[15] Hautiere M, Vivier D, Pineau D, Denis C, Kereselidze D, Herbet A (2023). ImmunoPET imaging-based pharmacokinetic profiles of an antibody and its Fab targeting endothelin A receptors on glioblastoma stem cells in a preclinical orthotopic model. Eur J Nucl Med Mol Imaging.

[16] Harland SP, Kuc RE, Pickard JD, Davenport AP (1995). Characterization of endothelin receptors in human brain cortex, gliomas, and meningiomas. J Cardiovasc Pharmacol.

[17] Harland SP, Kuc RE, Pickard JD, Davenport AP (1998). Expression of endothelin(A) receptors in human gliomas and meningiomas, with high affinity for the selective antagonist PD156707. Neurosurgery.

[18] Egidy G, Eberl LP, Valdenaire O, Irmler M, Majdi R, Diserens A-C (2000). The endothelin system in human glioblastoma. Lab Invest.

[19] Tsutsumi K, Niwa M, Kitagawa N, Yamaga S, Anda T, Himeno A (1994). Enhanced expression of an endothelin ETA receptor in capillaries from human glioblastoma: a quantitative receptor autoradiographic analysis using a radioluminographic imaging plate system. J Neurochem.

[20] Bowman RL, Wang Q, Carro A, Verhaak RGW, Squatrito M (2017). GlioVis data portal for visualization and analysis of brain tumor expression datasets. Neuro Oncol.

[21] Lange F, Kaemmerer D, Behnke-Mursch J, Brück W, Schulz S, Lupp A (2018). Differential somatostatin, CXCR4 chemokine and endothelin A receptor expression in WHO grade I-IV astrocytic brain tumors. J Cancer Res Clin Oncol.

[22] Lupp A, Mann A, Heeb A, Kaemmerer D, Sänger J, Evert M (2015). Reassessment of endothelin receptor A expression in normal and neoplastic human tissues using the novel rabbit monoclonal antibody UMB-8. Peptides.

[23] Vivier D, Hautière M, Pineau D, Dancer P-A, Herbet A, Hugnot J-P (2023). Synthesis and preclinical fuorescence imaging of dually functionalized antibody conjugates targeting endothelin receptor-Positive Tumors. Bioconjugate Chem.

[24] Durocher Y, Perret S, Kamen A (2002). High-level and high-throughput recombinant protein production by transient transfection of suspension-growing human 293-EBNA1 cells. Nucleic Acids Res.

[25] Li MZ, Elledge SJ (2012). SLIC: a method for sequence- and ligation-independent cloning. In: Peccoud J, Ed. Gene Synthesis: Methods and Protocols. Totowa, NJ: Humana Press.

[26] Herbet A, Costa N, Leventoux N, Mabondzo A, Couraud J-Y, Borrull A (2018). Antibodies targeting human endothelin-1 receptors reveal different conformational states in cancer cells. Physiol Res.

[27] Guichet P-O, Bieche I, Teigell M, Serguera C, Rothhut B, Rigau V (2013). Cell death and neuronal differentiation of glioblastoma stem-like cells induced by neurogenic transcription factors. Glia.

[28] Bouleau A, Nozach H, Dubois S, Kereselidze D, Chevaleyre C, Wang C-I (2022). Optimizing immuno-PET imaging of tumor PD-L1 expression: pharmacokinetic, biodistribution, and dosimetric comparisons of 89Zr-labeled anti-PD-L1 antibody formats. J Nucl Med.

[29] Shah R, Vattoth S, Jacob R, Manzil FFP, O'Malley JP, Borghei P (2012). Radiation necrosis in the brain: imaging features and differentiation from tumor recurrence. RadioGraphics.

[30] Verger A, Langen K-J (2017). PET imaging in glioblastoma: use in clinical practice. In: De Vleeschouwer S, Ed. Glioblastoma. Brisbane (AU): Codon Publications.

[31] Kasten BB, Jiang K, Cole D, Jani A, Udayakumar N, Gillespie GY (2020). Targeting MMP-14 for dual PET and fluorescence imaging of glioma in preclinical models. Eur J Nucl Med Mol Imaging.

[32] Juthani R, Madajewski B, Yoo B, Zhang L, Chen P-M, Chen F (2020). Ultrasmall core-shell silica nanoparticles for precision drug delivery in a high-grade malignant brain tumor model. Clin Cancer Res.

[33] Shi X, Xu P, Cao C, Cheng Z, Tian J, Hu Z (2022). PET/NIR-II fluorescence imaging and image-guided surgery of glioblastoma using a folate receptor α-targeted dual-modal nanoprobe. Eur J Nucl Med Mol Imaging.

[34] Yang J, Zhao C, Lim J, Zhao L, Tourneau RL, Zhang Q (2021). Structurally symmetric near-infrared fluorophore IRDye78-protein complex enables multimodal cancer imaging. Theranostics.

[35] Davenport AP, Hyndman KA, Dhaun N, Southan C, Kohan DE, Pollock JS (2016). Endothelin. Barker EL, Ed. Pharmacol Rev.

[36] Arvanitis CD, Ferraro GB, Jain RK (2020). The blood-brain barrier and blood-tumour barrier in brain tumours and metastases. Nat Rev Cancer.

[37] Sarkaria JN, Hu LS, Parney IF, Pafundi DH, Brinkmann DH, Laack NN (2018). Is the blood-brain barrier really disrupted in all glioblastomas? A critical assessment of existing clinical data. Neuro Oncol.

[38] Chevaleyre C, Novell A, Tournier N, Dauba A, Dubois S, Kereselidze D (2023). Efficient PD-L1 imaging of murine glioblastoma with FUS-aided immunoPET by leveraging FcRn-antibody interaction. Theranostics.

[39] Abdul Y, Karakaya E, Chandran R, Jamil S, Ergul A (2022). Endothelin A (ETA) receptors contribute to senescence of brain microvascular endothelial cells. Can J Physiol Pharmacol.

[40] Latorraca NR, Venkatakrishnan AJ, Dror RO (2017). GPCR dynamics: structures in motion. Chem Rev.

