# Supplementary material for: Preoperative PET imaging and fluorescence-guided surgery of human glioblastoma using dual-labeled antibody targeting ETA receptors in a preclinical mouse model: A theranostic approach
Source: Theranostics. 2024 Sep 30;14(16):6268–80. doi: 10.7150/thno.98163 (PMC11488107; doi:10.7150/thno.98163)
Supplement: Supplementary file 1 — Supplementary materials and methods, figures, table, and video legends. [file thnov14p6268s1.pdf]

Supporting information: 'Preoperative PET imaging and fluorescence-guided surgery of human glioblastoma using dual-labeled antibody targeting ETA receptors in a preclinical mouse model: A theranostic approach'

Marie Hautiere [1,4]*, Delphine Vivier [2]*, Paul Dorval [3], Donovan Pineau [5], Dimitri Kereselidze [4], Caroline Denis [4], Amaury Herbet [1], Narciso Costa [1], Claire Bernhard [2], Victor Goncalves [2], Erwan Selingue[6], Benoit Larrat[6], Pierre-Alix Dancer [3], Jean Philippe Hugnot [5]**, Didier Boquet [1]**+, Charles Truillet [4]**+, Franck Denat [2]**+

[1] Université Paris-Saclay, CEA, INRAE, Médicaments et Technologies pour la Santé (MTS), SPI, Laboratoire d'Etude de l'Unité Neurovasculaire et Innovation Thérapeutique (LENIT), 91191 Gif-sur-Yvette, France

[2] Université de Bourgogne, ICMUB UMR CNRS 6302, Dijon, 21000, France

[3] Kaer Labs, 44300 Nantes, France

[4] Université Paris-Saclay, CEA, CNRS, Inserm, BioMaps, Orsay, 91401, France

[5] Université de Montpellier, IGF, INSERM U 1191 - CNRS UMR 5203, Montpellier, 34094, France

[6] Université Paris-Saclay, CEA, CNRS, NeuroSpin/BAOBAB, Gif sur Yvette, France

* The authors are joint first authors. ** The authors are joint last authors.

[+] Corresponding author: Franck Denat (MOMIP plateform, dual labeling: nuclear imaging and fluorescence) franck.denat@u-bourgogne.fr

+ Corresponding author: Charles Truillet (immunoPET imaging and optical imaging) charles.truillet@universite-paris-saclay.fr

+ Corresponding author: Didier Boquet (RA63 mAb properties and endothelin axis)

didier.boquet@cea.fr

MATERIALS AND METHODS SUPPLEMENTAL DATA

Antibody production

Synthesis of the MOMIP

General remarks:

All chemicals, unless otherwise noted, were acquired from Sigma-Aldrich, Acros Organics and Alfa Aesar and used without further purification. All water used was ultra-pure (>18.2 MΩcm-1).

Purifications by semi-preparative HPLC were performed on an UltiMate 3000 system Dionex (Thermo Scientific) equipped with an UV-visible detector, on the following columns: BetaBasic-18 column (Thermo Scientific) (5 µm, 150 Å) at 20 mL/min, or Hypersil GOLD column 100 x 10 (Thermo Scientific) (5 µm, 175 Å) at 3.5 mL/min, with HPLC grade eluents. Unless specified otherwise, purifications were performed using acetonitrile and water, both supplemented with 0.1% of trifluoroacetic acid. The fractions of interest were analyzed by HPLC-MS, pooled, concentrated under reduced pressure to remove organic solvents and freeze-dried.

High performance liquid chromatography (HPLC) analyses were performed on an UltiMate 3000 system Dionex (Thermo Scientific) equipped with a DAD detector and coupled to a low-resolution mass spectrometry detector MSQ Plus (Thermo Scientific) with an ESI source. Separation was achieved using an RP Kinetex™ column (Phenomenex) (2.6 µm, 100 Å, 50 Å, 50 x 2.1 mm) with HPLC quality solvents: A: H_2O + 0.1% formic acid (FA) and B: MeCN + 0.1% FA.

Analyses were performed with the following gradient program: 5% to 100% of B in 5 min, 100% B for 1.5 min, 100% to 5% B in 0.1 min and 5% B for 1.9 min, at a flow rate of 0.5 mL/min. NMR spectra were acquired on a Bruker NMR spectrometer 500 MHz Avance DRX. HRMS spectra were recorded on a high-resolution Orbitrap mass spectrometry (Exploris 240, Thermo Fisher Scientific) using an ESI source.

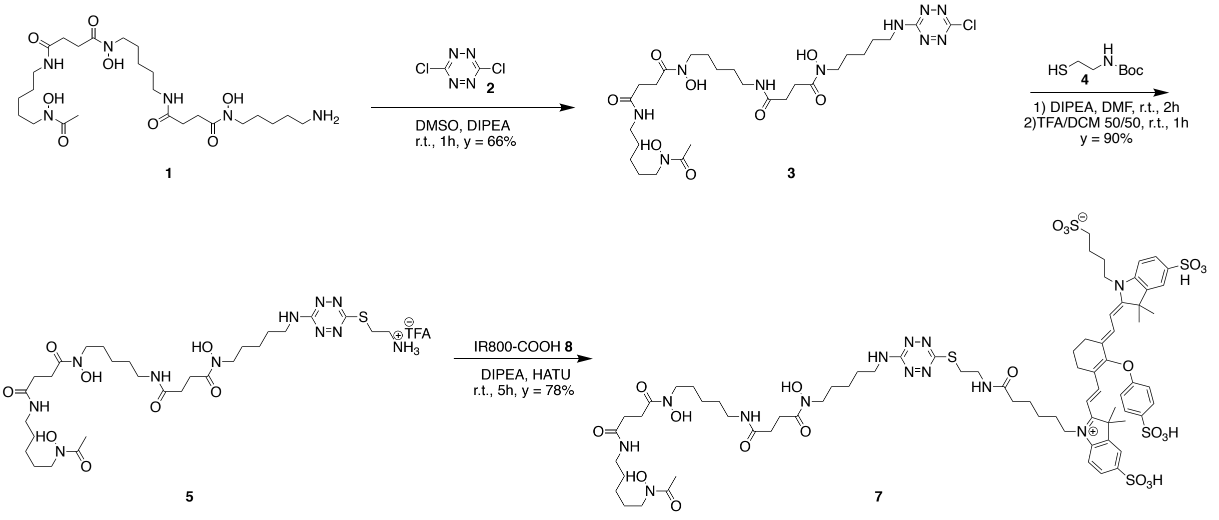

DFO-NH-Tz-Cl **3**

Desferrioxamine mesylate salt **1** (1 equiv., 229 µmol, 150 mg) was added to a solution of 3,6-dichloro-1,2,4,5-tetrazine **2** (1.2 equiv., 275 µmol, 41.3 mg) in dry DMSO (3 mL) with N,N-diisopropylethylamine (4 equiv., 915 µmol, 160 µL). The reaction mixture was stirred at room temperature for 1 h and purified on semi-preparative HPLC on a BetaBasic-18 column (A: H_2O 0.1% TFA, B: MeCN 0.1% TFA; with the

following gradient program: 2% of B for 8 min, 2 to 30% in 5 min, 30 % to 60% of B in 60 min, at a flow rate of 20 mL/min) to obtain an orange solid after lyophilization (m = 102 mg, y = 66%).

RP-HPLC-MS: tr = 4 min, m/z calculated for $C_{27}H_{48}ClN_{10}O_8$ $[M+H]^+$ 675.3, found 675.6.

^{1}H NMR (500 MHz, DMSO-*d*$_6$) δ 9.63 (s, 3H), 8.84 (t, *J* = 5.7 Hz, 1H), 7.77 (m, 2H), 3.50-3.46 (m, 4H), 3.36 (q, *J* = 6.7 Hz, 4H), 2.99 (q, *J* = 6.6 Hz, 4H), 2.57 (t, *J* = 7.4 Hz, 4H), 2.26 (t, *J* = 7.3 Hz, 4H), 1.96 (s, 3H), 1.64-1.56 (m, 2H), 1.56-1.44 (s, 6H), 1.42-1.34 (m, 4H), 1.33-1.28 (m, 2H), 1.26-1.16 (m, 4H). ^{13}C NMR (126 MHz, DMSO-*d*$_6$) δ 172.1 (C), 171.4 (C), 170.3 (C), 161.4 (C), 158.5 (C), 47.1 (CH_2), 46.9 (CH_2), 40.7 (CH_2), 38.5 (CH_2), 30.0 (CH_2), 28.9 (CH_2), 27.7 (CH_2), 27.6 (CH_2), 26.1 (CH_2), 23.5 (CH_2), 23.5 (CH_2), 20.4 (CH_3).

DFO-NH-Tz-S-NH$_2$ **5**

To a solution of DFO-NH-Tz-Cl **3** (1 equiv., 89 μmol, 60 mg) in DMF (2 mL) and DIPEA (8 equiv., 712 μmol, 124 μL) was added a solution of tert-butyl (2-mercaptoethyl)carbamate **4** (1.2 equiv., 107 μmol, 19 mg) in DMF (2.5 mL). The resulting orange solution was stirred at room temperature. After 2 h, the product was precipitated from diethyl ether, redissolved into 2 mL of CH_2Cl_2 and 2 mL of TFA and the resulting solution was stirred at room temperature for 1 h. After concentration under vacuum the mixture was purified by semi-preparative HPLC on a BetaBasic-18 column (A: H_2O 0.1% TFA, B: MeCN 0.1% TFA; with the following gradient program: 2% of B for 8 min, 2 to 20% in 5 min, 20 % to 55% of B in 40 min, at a flow rate of 20 mL/min) to afford an orange powder (m = 58 mg, y = 90%).

RP-HPLC-MS: t_r = 3.75 min, *m/z* calculated for $C_{29}H_{54}N_{11}O_8S$ $[M+H]^+$ 716.4, found 716.6.

^{1}H NMR (500 MHz, DMSO-*d*$_6$) δ 9.67-9.61 (m, 3H), 8.48-8.40 (m, 1H), 7.91 (s, 3H), 7.75-7.66 (m, 2H), 3.43-3.37 (m, 8H), 3.31-3.25 (m, 2H), 3.14-3.04 (m, 2H), 2.96-2.87 (m, 4H), 2.50 (t, *J* = 7.3 Hz, 4H), 2.19 (t, *J* = 7.3 Hz, 4H), 1.89 (s, 3H), 1.58-1.49 (m, 2H), 1.49-1.37 (s, 6H), 1.35-1.26 (m, 4H), 1.25-1.20 (m, 2H), 1.19-1.09 (m, 4H). ^{13}C-NMR (126 MHz, D_2O) δ = 172.0 (C), 171.4 (C), 170.2 (C), 163.2 (C), 161.0 (C), 47.1 (CH_2), 46.8 (CH_2), 40.4 (CH_3), 38.5 (CH_2), 38.3 (CH_2), 29.9 (CH_2), 29.9 (CH_2), 28.8 (CH_2), 27.9 (CH_2), 27.6 (CH_2), 27.6 (CH_2), 27.4 (CH_2), 26.1 (CH_2), 23.5 (CH_2), 20.4 (CH_2) ppm.

DFO-NH-Tz-S-IR800 **7**

To a solution of IRDye800CW **6** (1 equiv., 6.35 µmol, 6.9 mg) and HATU (1.1 equiv., 7.0 µmol, 2.7 mg) in DMF (250 µL) with DIPEA (1.1 equiv., 7.0 µmol, 1.2 µL) was added DFO-NH-Tz-S-NH$_2$ **5** (1.1 equiv., 7.0 μmol, 5.0 mg) in DMF (250 µL). The green solution was stirred at room temperature for 5 h. After concentration under vacuum, the mixture was purified by semi-preparative HPLC on an Hypersil Gold column (A: TEAB 50 mM, B: MeCN with the following gradient program: 2% of B for 8 min, 2 to 20% in 5 min, 20 % to 55% of B in 50 min, at a flow rate of 3.5 mL/min) to give a blue/green powder (m = 8.4 mg, y = 78%). RP-HPLC-MS: t_r = 3.6 min., *m/z* calculated for $C_{75}H_{106}N_{13}O_{22}S_5$ $[M+H]^+$ 1700.6, found 1700.9, *m/z* calculated for $[M+2H]^{2+}$ 850.8, found 851.3. HRMS (Fig S1): *m/z* calculated for $C_{75}H_{106}N_{13}O_{22}S_5$ $[M+H]^+$ 1700.61734, found 1700.62481, *m/z* calculated for $[M+2H]^{2+}$ 850.81231, found 850.81535, *m/z* calculated for $[M-H]^-$ 1698.60279, found 1698.60632, *m/z* calculated for $[M-2H]^{2-}$ 848.79776, found 848.80066.

Preparation of the bimodal conjugate

General remarks

Each of the following reactions were followed by reverse phase HPLC (Vanquish, Thermo Fisher Scientific) coupled to a high-resolution Orbitrap mass spectrometry (Exploris 240, Thermo Fisher Scientific) using an ESI source (positive mode). HPLC: A MabPac RP column (2.1 mm x 100 mm, 4µm, Thermo Fisher Scientific) was used at 80°C (denaturing conditions), with a flow rate of 0.5 mL/min and a gradient 20-45% B in 6 min (eluents A: H_2O + 0.02% TFA + 0.08% FA and B: ACN + 0.02% TFA + 0.08% FA). Mass: Full scan data were acquired at resolving power 30000 at m/z 200 in the m/z range 1000–4 000. Data were analyzed with FreeStyle and BioPharma Finder 4.1 software (both Thermo Fisher Scientific). High-resolution mass spectra were deconvoluted using the Xtract function for IDES fragments (25 kDA) and ReSpect function for DTT reduced fragments (50 kDa) and intact antibody (150 kDa). Drug-to-antibody ratio was determined by comparison of native and conjugated antibody masses.

ss-axiRA63-TCO: To a solution of axiRA63 – N297Q-xiRA63 – (750 µg, 45 µL at 16.7 mg/mL in PBS) was added TCO-PEG_4-NH_2 (60 equiv., 5.11 µL at 60mM in PBS), MTGase (5U/mg of antibody, 3.8 U, 28.1 µL) and 22 µL of phosphate buffered saline (PBS, pH 7.4) to adjust the antibody concentration to 7.5g/L. The reaction was incubated at 25 °C for 2 h and then purified via size exclusion chromatography (Sephadex G-25 M, PD-10 column, GE Healthcare) and concentrated using centrifugal filter units with a 50,000 Da molecular weight cut off (Amicon^{TM} Ultra 2 mL Centrifugal Filtration Units, Millipore Corp., Billerica, MA) and phosphate buffered saline (PBS, pH 7.4) to obtain 721 µg of ss-axiRA63-TCO. The degree of labeling was measured by mass spectrometry (DOL = 4).

axiRA63-MOMIP: To a solution of ss-axiRA63-TCO (550 µg, 50 µL at 11.1 mg/mL in PBS) was added the MOMIP DFO-Tz-IR800 (10 equiv. *per* accessible TCO, 5.0 µL at 30 mmol/L in DMSO) and the antibody concentration was adjusted to 5 g/L by adding 56 µL of phosphate buffered saline (PBS, pH 7.4). The reaction mixture was stirred at room temperature overnight and purified by Protein A affinity chromatography (Hitrap Mabselect Xtra column,Cytiva) to give 228 µg of axiRA63-MOMIP. The degree of labeling was measured by UV spectroscopy (DOL = 3.1 ± 0.1) and mass spectrometry (DOL = 2.5).

Degree of labeling (DOL) determination by UV-Vis spectrophotometry

The fluorophore-antibody ratio was calculated using the integral absorption to account for dye aggregation, as reported in the literature [1]. Specifically, the following formula was used:

$$DOL_{MOMIP/mAb} = \frac{F_{550-900} \times \varepsilon_{mAb}}{\varepsilon_{F550-900} \times (A_{280} - F_{550-900} \times CF_{280})}$$

with $F_{550\text{-}900}$, the integral of the absorption band between 550 and 900 nm,

$\varepsilon_{F550\text{-}900}$ = 13,600,000 nm.cm.L.mol^{-1}, the integral absorption coefficient of IRDye800CW,

A_{280}, the absorption at 280 nm,

ε_{mAb} = 206,000 cm.L.mol^{-1}, the extinction coefficient of the antibody at 280 nm,

CF_{280} = 0.0019 nm^{-1}, the correction factor for the absorbance of the dye at 280 nm.

$\varepsilon_{F550-900}$ and CF_{280} were calculated based on the absorption spectrum of a 30 µM solution of MOMIP in PBS pH 7.4.

Assessment of the [^{89}Zr]Zr-axiRA63-MOMIP antibody stability by iTLC.

The *in vivo* [^{89}Zr]Zr-axiRA63-MOMIP stability was evaluated at 48 h p.i. by iTLC. In this context, intracardiac blood sampling (500 µL to 1 mL of blood) was performed on 5 mice. Plasma was obtained by centrifuging the collected blood for 10 min at 1000g. Subsequently, 20 µL of plasma was applied on iTLC-SG glass microfiber papers impregnated with silica gel (Agilent Technologies). After migration using a citric acid solution with 5% acetonitrile (20 mM, pH 4.9–5.1) as the mobile phase, the iTLC was divided into two pieces: one containing the plasma solution deposit and the other the migration front. The activity of the two ends of the iTLC was read with a gamma counter (Cobra gamma counter, Packard). (Supplemental Figure 6).

SUPPLEMENTARY FIGURES

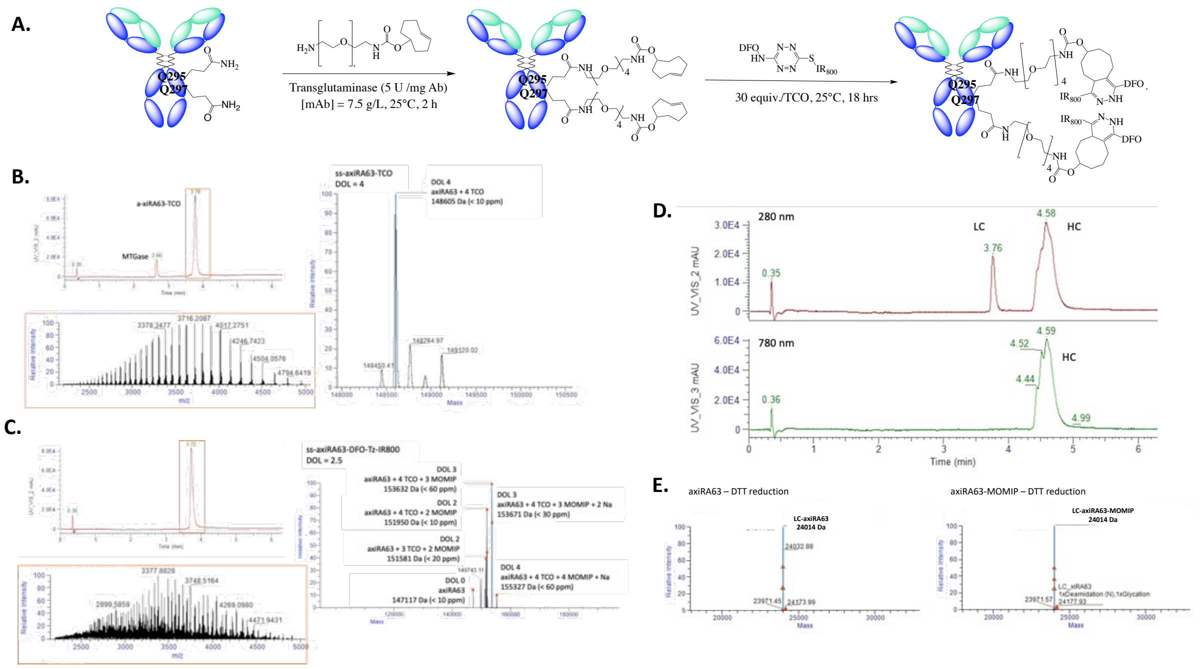

__Figure S1: Characterization of the axiRA63-MOMIP conjugate. A.__ Synthesis of the immunoconjugate axiRA63-MOMIP. __B.__ axiRA63-TCO characterization by liquid chromatography (UV chromatogram at 280 nm upper left) and non deconvoluted (bottom left) or deconvoluted mass spectrum (right). __C.__ axiRA63-MOMIP characterization by liquid chromatography (UV chromatogram at 280 nm upper left) and non deconvoluted (bottom left) or deconvoluted (right) mass spectrum. Errors between calculated and measured masses were less than 10 ppm, unless specified otherwise. __D.__ Liquid chromatography of reduced axiRA63-MOMIP (UV chromatogram at 280 and 780 nm) and __E.__ deconvoluted mass spectra of axiRA63 and axiRA63-MOMIP LC fragments confirming the heavy chain site-specific modification.

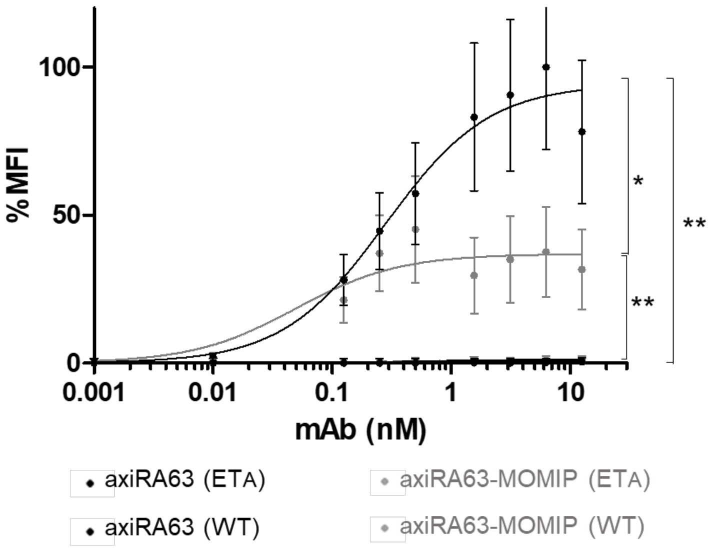

	axiRA63	axiRA63-MOMIP
**Bmax (%MFI)**	94.37 ± 0.12	36.95 ± 0.06
**Apparent Kd (nM)**	0.28 ± 0.002	0.05 ± 0.001

Figure S2: In vitro binding properties of axiRA63-MOMIP by flow cytometry. *Binding curves for xiRA63-N297Q antibody (solid black line) and axiRA63-MOMIP (grey solid line) on CHO-ETA (solid lines) and CHO-WT cells (dotted line). Data are presented as mean value ± standard deviation; MFI: Median Fluorescence Intensity. Statistical comparisons were made using paired two-tailed Student's t-test (** $p < 0.01$; * $p < 0.05$; ns: no significant difference).*

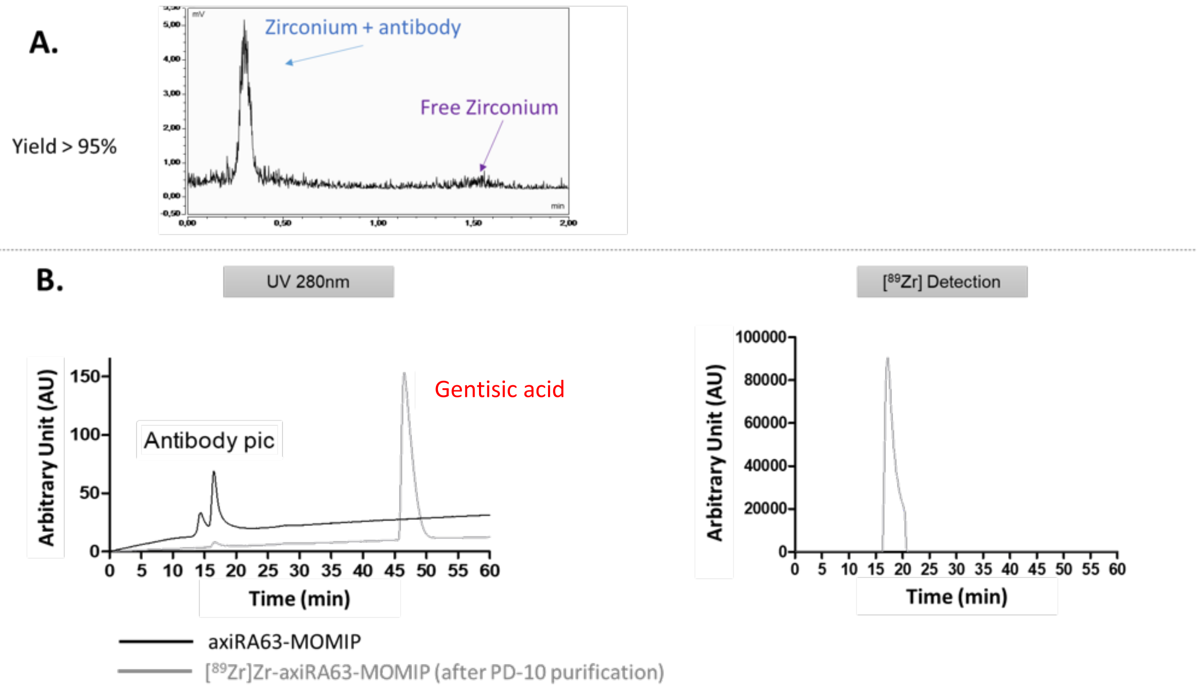

Figure S3: [^{89}Zr]-Zr-axiRA63-MOMIP radiolabeling assessment by iTLC (A) and HPLC (B).

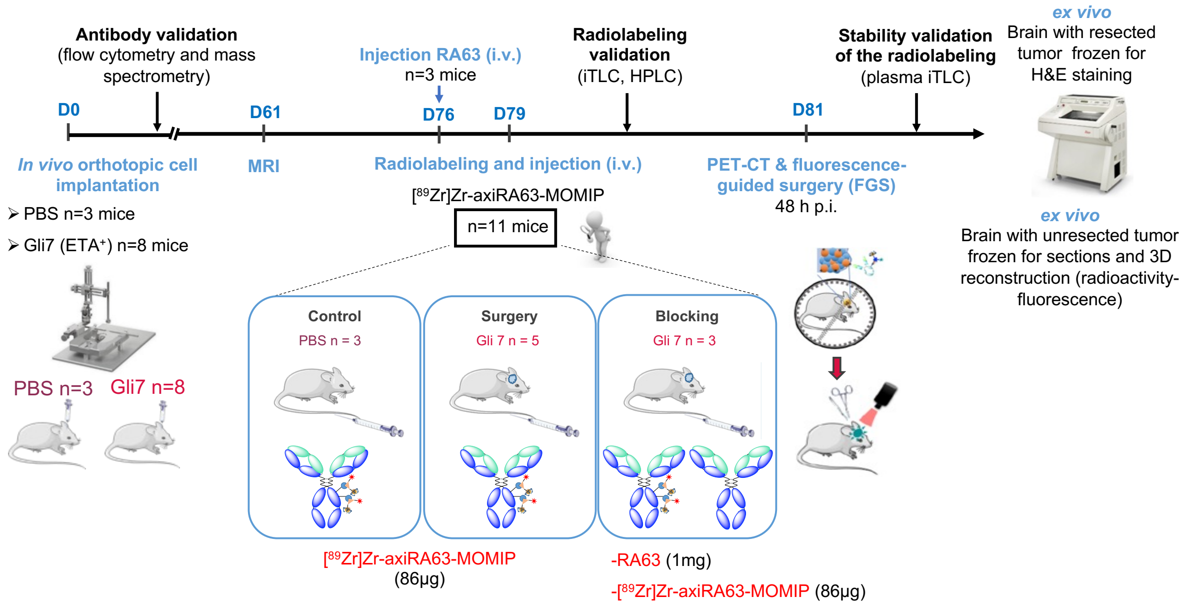

*Figure S4**: Implemented protocol for human GBM fluorescence-guided tumor resection pre-localized by immunoPET.** Mice were MRI imaged 61 days after tumor cells implantation. On day 76 post-implantation, the blocking group received an intravenous bolus of RA63. 3 days later (day 79 post-implantation), the three groups were injected with [^{89}Zr]Zr-axiRA63-MOMIP. All mice were imaged by immunoPET 48 h post injection of [^{89}Zr]Zr-axiRA63-MOMIP. After PET imaging, intracardiac blood collection was performed to validate the antibody in vivo stability. Next, fluorescence-guided surgery was performed on mice using the KIS 800 system (Kaer Labs). One of the brains with resected tumor was frozen to check the presence of residual tumor cells using H&E labeling. Another brain from the surgery group was frozen with the unresected tumor to assess the colocalization of the fluorescence and radioactivity signals.*

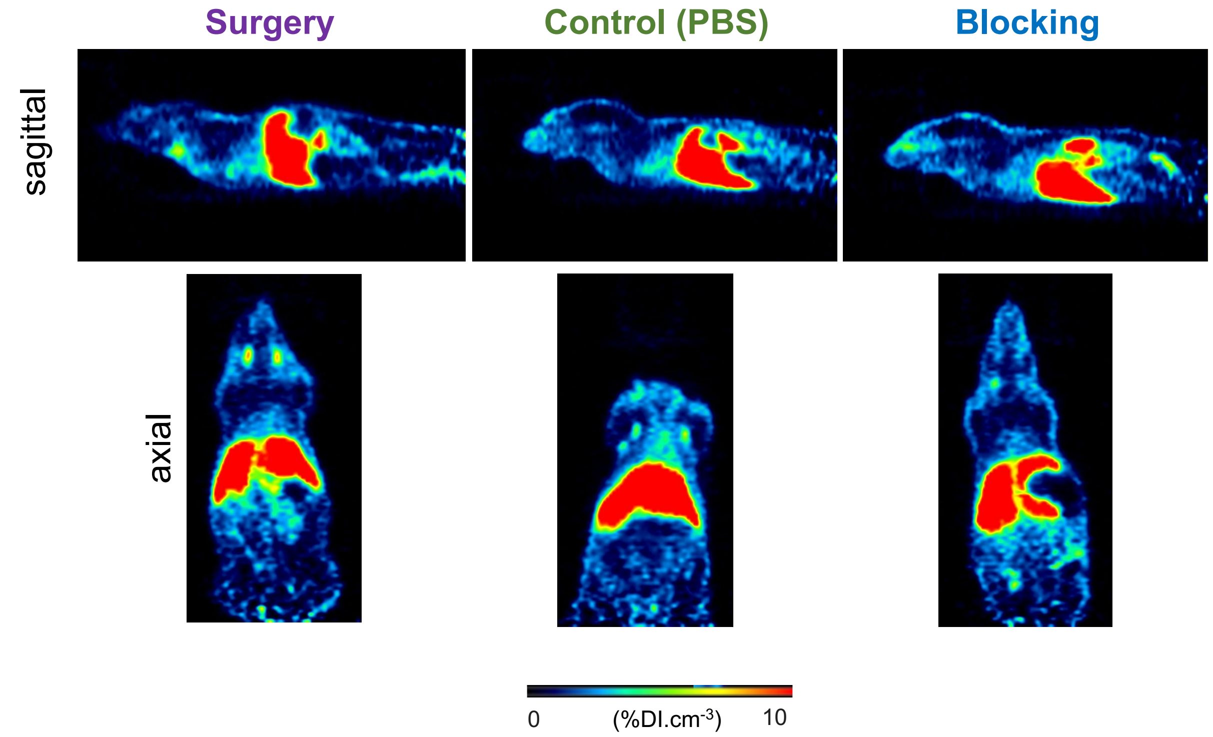

*Figure S5: **Qualitative biodistribution of the [^{89}Zr]Zr-axiRA63-MOMIP conjugate at 48 h p.i.** in the different groups of mice.*

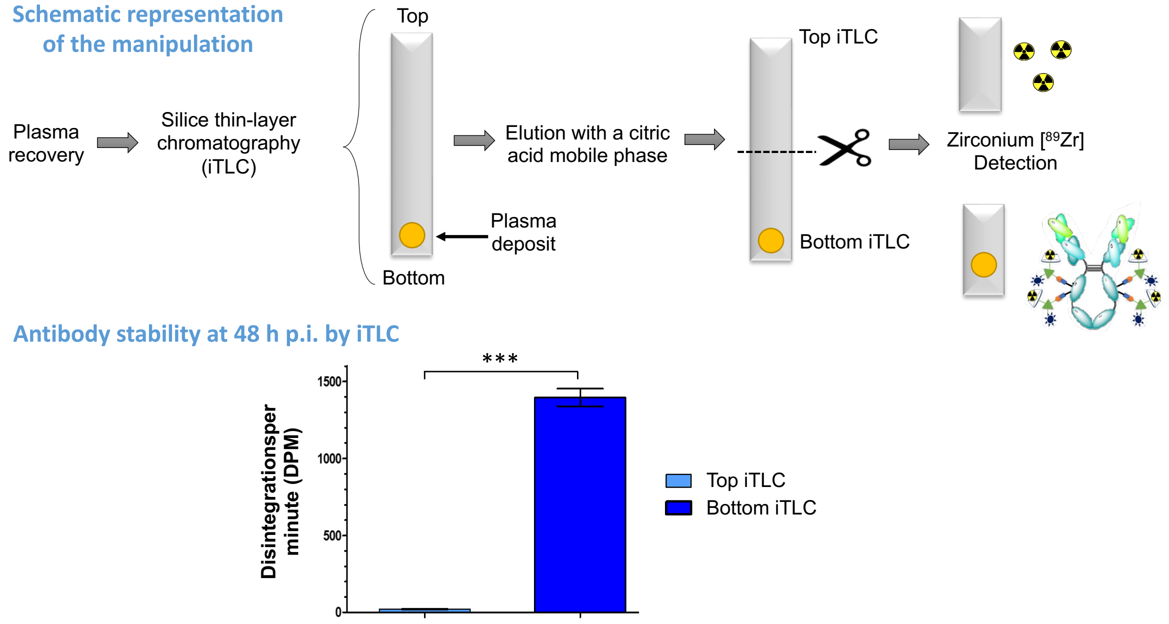

Figure S6 : Assessment of the [^{89}Zr]Zr-axiRA63-MOMIP antibody stability by iTLC.

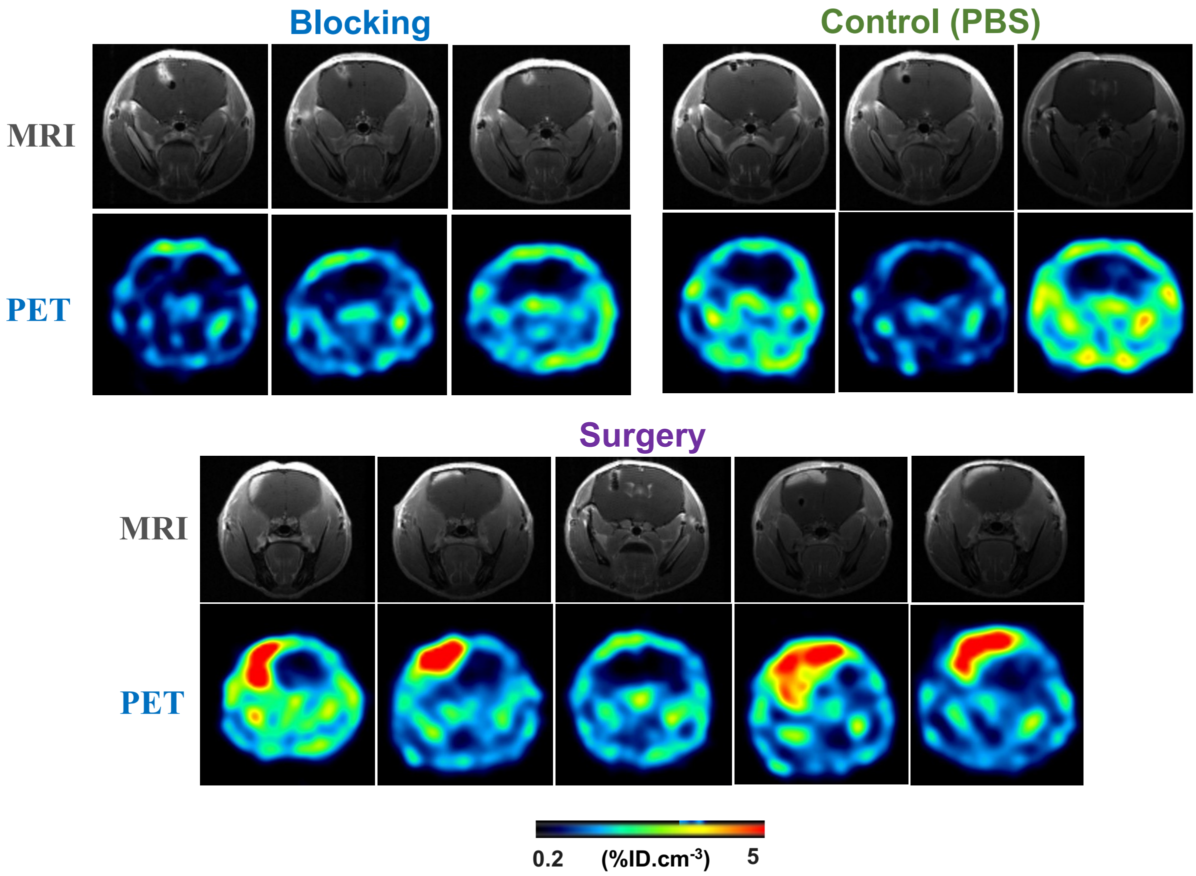

Figure S7: Coronal section of the brain tumor PET imaging at 48 h p.i.

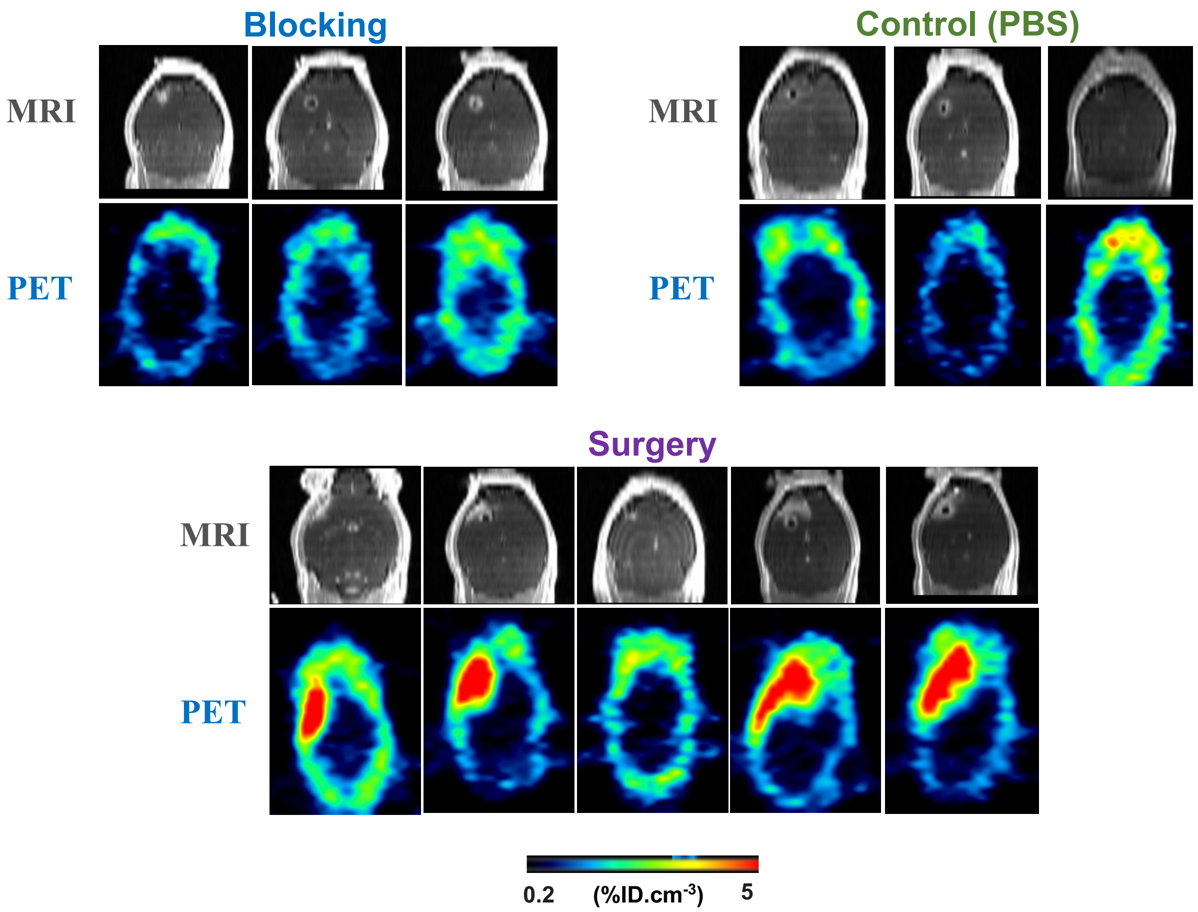

Figure S8: Axial section of the brain tumor PET imaging at 48 h p.i.

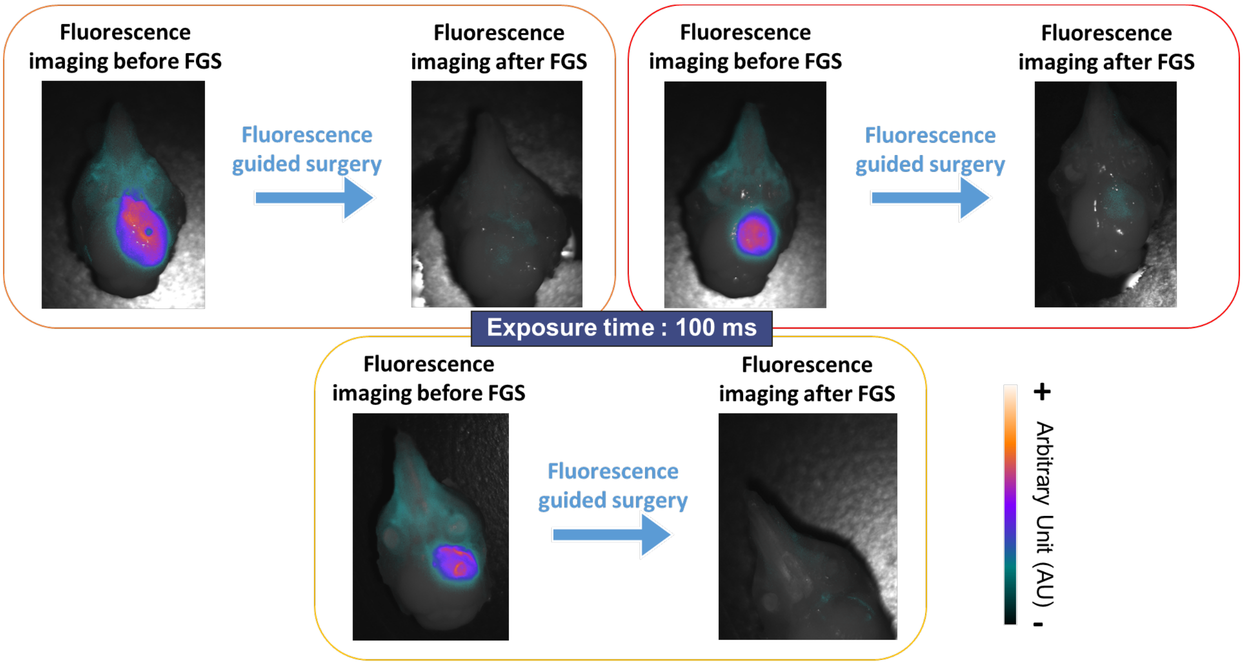

Figure S9: Fluorescence guided surgery of the brain tumor Gli7 ETA^{+} mediated by [^{89}Zr]Zr-axiRA63-MOMIP conjugate 48 h p.i.

SUPPLEMENTARY TABLE

Table S1: Overview table of the PET experiments on mice bearing human GBM tumor injected with [^{89}Zr]Zr-axiRA63-MOMIP. *Data are presented as mean ± SD.*

Tumor model	Antibody label [^{89}Zr]	Specific activity (MBq/nM)	Injected activity (MBq)	Injected quantity (nmol)	Number of mice
Gli7	[^{89}Zr]Zr-xiRA63-MOMIP-IR800	0.008 ± 0.0006	4.3 ± 0.1	555 ± 12.8	11

SUPPLEMENTARY VIDEOS

Video S1: Overlay_Surgey_mouse3

Video S2: Overlay_Surgery_mouse5

Video S3: Overlay_Surgery_mouse9

1. Grabolle M, Brehm R, Pauli J, Dees FM, Hilger I, Resch-Genger U. Determination of the Labeling Density of Fluorophore–Biomolecule Conjugates with Absorption Spectroscopy. Bioconjugate Chem. 2012; 23: 287–92.
